# Supplementary material for: First-in-human phase I dose escalation trial of the first-in-class tumor microenvironment modulator VT1021 in advanced solid tumors
Source: Commun Med (Lond). 2024 Jan 13;4:10. doi: 10.1038/s43856-024-00433-x (PMC10787778; doi:10.1038/s43856-024-00433-x)
Supplement: Supplementary file 1 — Description of Additional Supplementary Files [file 43856_2024_433_MOESM1_ESM.docx]

**Description of Additional Supplementary Files**

**File Name:** Supplementary Data 1

**Description:** Source data supporting Figure 2, Figure 4 and Supplementary Figure 1.

**File Name:** Supplementary Data 2

**Description:** Source data for supplementary Figure 1
