# Supplementary material for: First-in-human phase I dose escalation trial of the first-in-class tumor microenvironment modulator VT1021 in advanced solid tumors
Source: Commun Med (Lond). 2024 Jan 13;4:10. doi: 10.1038/s43856-024-00433-x (PMC10787778; doi:10.1038/s43856-024-00433-x)
Supplement: Supplementary file 2 — Supplementary Information [file 43856_2024_433_MOESM2_ESM.pdf]

Supplementary Information

Supplementary Notes

Supplementary Figure 1

VT1021 reprograms the tumor microenvironment by increasing M1:M2 macrophage ratio.
a Graph of dot plots with error bars depicting M1:M2 macrophage ratio in pre- vs on-study biopsy of a patient with ovarian cancer, the line with error bars indicating the mean and standard error of the mean (SEM), $p=0.0228$, $n=3$ for both pre- and on-treatment samples; **b** Image of metal-ion immunostaining (MIBI) of CD68 and iNOS (M1) and CD68 and CD163 (M2); **c**
Graph of dot plots with error bars depicting quantitation of TSP-1 expression in pre- vs on-study biopsy as determined by MIBI, the line with error bars indicating the mean and SEM, $p<0.0001$, $n=15152$ for pre-treatment, and $n=7791$ for on-treatment; **d** Image of MIBI of TSP-1. Bar denotes 144 µm. iNOS: inducible nitric oxide synthase.

Statistical analysis was performed with Graphpad Prism 9.3.1, p values were calculated by unpaired two sample t-test, graphs of a point with error bars are used to indicate the average values and standard error of the mean (SEM).

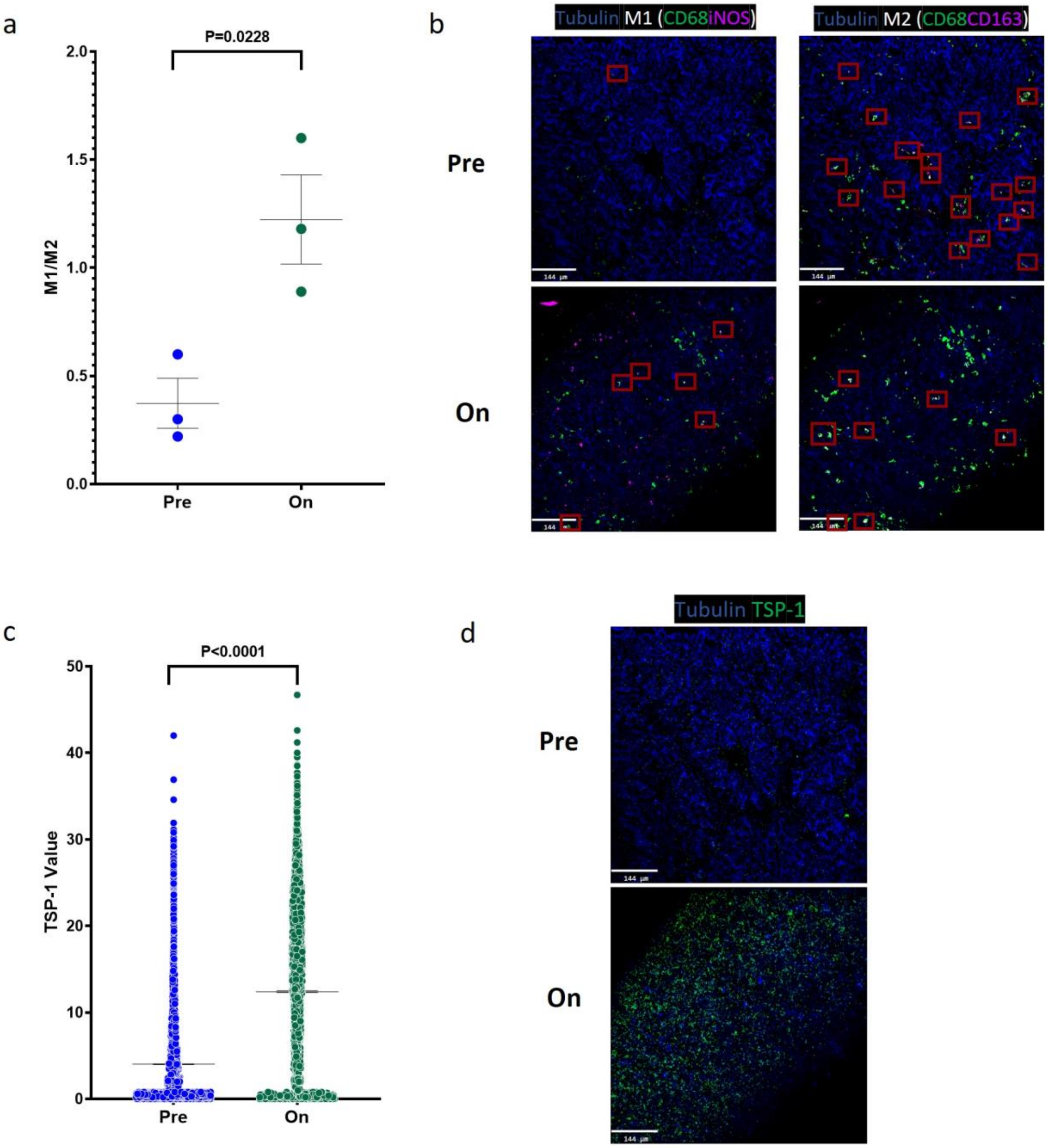

a
P=0.0228
M1/M2
2.0
1.5
1.0
0.5
0.0
Pre
On
b
Tubulin M1 (CD68iNOS)
Tubulin M2 (CD68CD163)
Pre
On
144 µm
c
P<0.0001
TSP-1 Value
50
40
30
20
10
0
Pre
On
d
Tubulin TSP-1
Pre
On
144 µm

Supplementary Methods

VT1021-01 Study Protocol

A Phase 1 Study Evaluating the Safety, Pharmacology, and Preliminary Activity of VT1021 in Patients with Advanced Solid Tumors

Protocol #VT1021-01

**Sponsor**	Vigeo Therapeutics, Incorporated<br>86A Sherman St<br>Cambridge, MA 02140<br>Phone: 617-945-0385
**IND Number**	133157
**Investigational Product**	VT1021
**Medical Monitor**	Lou Vaickus, MD
**Protocol Version Date**	08 April 2020
**Protocol Version**	4.0

Version History:

Protocol Version	Date Finalized
1.0	16 June 2017
1.1	21 July 2017
1.2	18 September 1017
1.3	13 April 2018
1.4	30 November 2018
2.0	5 April 2019
3.0	10 September 2019
4.0	08 April 2020

CONFIDENTIALITY STATEMENT

This document and the information it contains is confidential and the proprietary property of Vigeo Therapeutics. The information is not to be disclosed or transmitted to any party without the written approval of Vigeo Therapeutics or its agents, and any such unauthorized use or disclosure is expressly prohibited.

PROTOCOL SIGNATURES

Jing Watnick, PhD<br>President and CEO	Date
Lou Vaickus, MD<br>Medical Monitor	Date

STUDY PERSONNEL

**Medical Monitor**	Lou Vaickus, MD<br>86A Sherman St.<br>Cambridge, MA 02140<br>Office: 617-313-7730<br>Mobile: 857-919-2493<br>Email: lou.vaickus@vigeotherapeutics.com

I have reviewed the protocol entitled “A Phase 1 Study Evaluating the Safety, Pharmacology, and Preliminary Activity of VT1021 in Patients with Advanced Solid Tumors” (Protocol VT1021-01) and agree that it contains all the information necessary to conduct the study as required. I will conduct the study in accordance with the principles of ICH Good Clinical Practice and the Declaration of Helsinki.

I will maintain as confidential all written and verbal information provided to me by the Sponsor, including but not limited to, the protocol, case report forms, Investigators’ brochure, material supplied at Investigator meetings, minutes of teleconferences, etc. Such material will only be provided as necessary to site personnel involved in the conduct of the study, the IRB or IEC, or local regulatory authorities.

I will obtain written informed consent from each prospective study patient or each prospective study patient’s legal representative prior to conducting any protocol-specified procedures. The consent form used will have the approval of the local IRB or IEC.

I will maintain adequate source documents and record all observations, study drug, and procedures pertinent to study patients in their medical records. I will accurately complete the case report forms supplied by the Sponsor in a timely manner. I will ensure that my facilities and records will be available for inspection by representatives of the Sponsor, the local IRB or IEC or local regulatory authorities. I will ensure that I and my staff are available to meet with representatives of the Sponsor during regularly scheduled monitoring visits.

I will notify the Sponsor within 24 hours of any serious adverse events. Following this notification, a written report describing the serious adverse event will be provided to the Sponsor as soon as possible, but no later than three days following the initial notification.

____

Investigator’s Name (Print)

____

Investigator’s Signature

____

Date

TABLE OF CONTENTS

1. GLOSSARY 14
2. PROTOCOL SYNOPSIS 18
3. INTRODUCTION 26
3.1. Study Rationale 26
3.2. Thrombospondin-1 26
3.3. The Role of Prosaposin in Tsp-1 Production 27
3.4. VT1021 Pre-Clinical Pharmacology Studies 28
3.4.1. Ovarian Cancer Models 28
3.4.2. Pancreatic Cancer Model 29
3.4.3. Melanoma Model 30
3.4.4. Triple-Negative Breast Cancer Model 31
3.5. VT1021 Nonclinical Toxicology 34
3.6. Rationale for Human Starting Dose and Dose Escalation Schedule 35
4. OBJECTIVES 36
4.1. Primary Objective – Dose Escalation Phase 36
4.2. Primary Objective – Dose Expansion Phase 36
4.3. Secondary Objectives 36
4.4. Exploratory Objectives 36
5. STUDY DESIGN 37
5.1. Dose Escalation Phase 37
5.2. Dose Expansion Phase 38
5.3. Determination of Maximum Tolerated Dose and Recommended Phase 2 Dose 38
5.3.1. Maximum Tolerated Dose 38
5.3.2. Recommended Phase 2 Dose 39
6. Enrollment 40
6.1. Sample Size 40
6.2. Study Sites 40
6.3. Inclusion Criteria 40
6.4. Exclusion Criteria 42

6.5. Patient Discontinuation ... 43
6.6. Patient Replacement ... 44
7. VT1021 TREATMENT ... 45
7.1. Administration and Description ... 45
7.1.1. Premedication Regimen ... 45
7.2. Storage, Handling, Packaging, and Labeling ... 46
7.3. Duration of Study Drug ... 46
7.4. Dosing Schedules ... 46
7.4.1. Dose Escalation Phase ... 46
7.4.2. Dose Expansion Phase ... 46
7.5. Dose Escalation/De-escalation Levels ... 46
7.6. Re-initiation of VT1021 after Dose Limiting Toxicity ... 47
7.7. Guidelines for Dose Modification ... 47
7.8. Management of Infusion-related Reactions ... 48
7.8.1. Reflex Testing for Infusion-related Reactions ... 48
7.8.2. Infusion-related Reactions Related to VT1021 ... 49
7.9. Study Drug Accountability ... 49
7.10. Concomitant Medications and Treatments ... 50
7.10.1. Permitted Concomitant Medications and Treatments ... 50
7.10.2. Prohibited Concomitant Medications and Treatments ... 51
7.11. Drug-Drug Interactions ... 51
8. STUDY PROCEDURES ... 52
8.1. Medical History ... 52
8.2. Safety Assessments ... 52
8.2.1. Physical Examination ... 52
8.2.2. Vital Signs ... 52
8.2.3. ECOG/Karnofsky PS ... 52
8.2.4. Electrocardiograms ... 52
8.3. Laboratory Parameters ... 53
8.4. Pharmacokinetic Parameters ... 54

8.4.1. Dose Escalation Cohorts 54
8.4.2. Dose Expansion Cohort 55
8.5. Pharmacodynamic Parameters 55
8.5.1. Dose Escalation Cohorts 56
8.5.2. Dose Expansion Cohorts 57
8.6. RECIST and iRECIST or RANO Assessment 58
8.6.1. Tumor Biomarker Assessment 59
8.7. Preliminary Efficacy Parameters 60
8.7.1. Best Overall Response 60
8.7.2. Duration of Response 60
8.7.3. Progression-Free Survival 60
9. SAFETY REPORTING 61
9.1. Definitions 61
9.1.1. Adverse Event 61
9.1.2. Suspected Adverse Reaction 61
9.1.3. Unexpected Adverse Event 61
9.1.4. Serious Adverse Event 61
9.1.5. Dose Limiting Toxicity 62
9.2. Grading of Adverse Events 63
9.3. Determination of Causality 63
9.4. Adverse Event Reporting 64
9.4.1. Expedited Reporting of Adverse Events 65
9.5. Adverse Event Follow Up 66
9.6. Pregnancy 66
10. STATISTICAL METHODS 67
10.1. Sample Size Determination 67
10.2. Study Populations 67
10.3. Statistical Analysis 67
10.3.1. General Considerations 67
10.3.2. Sample Size Determination 68

10.3.3. Patient Disposition, Demographics and Baseline Characteristics 68
10.3.4. Exposure to VT1021 68
10.3.5. Preliminary Efficacy Analyses 68
10.3.6. Safety Analyses 68
10.3.7. Interim Analyses 69
10.3.8. Pharmacokinetic, Pharmacodynamic and Biomarker Analyses 69
11. ETHICAL ASPECTS 70
11.1. Ethical Conduct of the Study and IRB Oversight 70
11.2. Informed Consent 70
11.3. Patient Confidentiality 70
12. STUDY ADMINISTRATION 72
12.1. Monitoring 72
12.2. Data Collection and Data Quality Assurance 72
12.3. Pre-Study Documents 72
12.4. Protocol Amendments 73
12.5. Records Retention 73
12.6. Reporting and Publications 74
12.7. Study Discontinuation 74
13. REFERENCES 76
14. APPENDICES 78

List of Tables

Table 1: VT1021 Dose Levels 47

Table 2: Pharmacokinetic Sample Collection - Dose Escalation Cohorts 54

Table 3: Pharmacokinetic Sample Collection – Five Patients in Each Dose Expansion Cohort Only 55

Table 4: Pharmacodynamic Sample Collection - Dose Escalation Cohort 57

Table 5: Pharmacodynamic Sample Collection – All Patients in Dose Expansion Cohorts 58

List of Figures

Figure 1: The effect of VT1021 on the growth of a human ovarian cancer xenograft in SCID mice........ 29

Figure 2: The effect of VT1021 on the growth of AsPc1 cells in SCID mice 30

Figure 3: Growth of B16 melanoma in syngeneic mice 31

Figure 4: Effect of VT1021 (10 mg/kg; QDx29) on MDA-MB-231 Human TNBC Tumor Growth in Athymic Nude Mice – Individual Animal Findings 32

Figure 5: Effects of VT1021 (25 mg/kg; QDx29) on MDA-MB-231 Human TNBC Tumor Growth in Athymic Nude Mice – Individual Animal Findings 32

Figure 6: Effect of VT1021 on Survival of C57BL6/J Immunocompetent Mice Inoculated with E0771 Breast Cancer Cells 33

Figure 7: Effect of VT1021 on E0771 Tumor Volume in C57BL6/J Immunocompetent Mice 34

List of Appendices

Appendix 1: Schedule of Assessments and Procedures........ 79

Appendix 2: The ECOG and Karnofsky Performance Scores........ 83

Appendix 3: RECIST 1.1 Criteria 85

Appendix 4: Description of the iRECIST Process for Assessment of Disease Progression........ 89

Appendix 5: CTCAE v5.0 Criteria 92

Appendix 6: The Declaration of Helsinki........ 93

Appendix 7: The RANO Criteria for Assessment of Glioblastoma Patients........ 94

1. GLOSSARY

Abbreviation	Definition
ALT	Alanine aminotransferase
ANC	Absolute neutrophil count
aPTT	Activated partial thromboplastin time
ASCO	American Society of Clinical Oncology
AST	Aspartate aminotransferase
AUC_{0-t}	Area under the curve from time zero to the last measurable concentration
$AUC_{0-\infty}$	Area under the curve from time zero to infinity
βHCG	Beta human chorionic gonadotrophin
BUN	Blood urea nitrogen
CFR	Code of Federal Regulations
C_{max}	Maximum dose concentration
CR	Complete response
CRA	Clinical research associate
CRF	Case report form
CRO	Clinical research organization
CT	Computed tomography
CTCAE	Common Terminology Criteria for Adverse Events
CTLA-4	Cytotoxic T-lymphocyte associated protein 4
DCR	Disease control rate
DLT	Dose limiting toxicity
DNA	Deoxyribonucleic acid
ECG	Electrocardiogram
ECOG	Eastern cooperative oncology group
eCRF	Electronic case report form
EDC	Electronic data capture
ELISA	Enzyme-linked immunosorbent assay
EOS	End of study
FACS	Fluorescence-activated cell sorting
FDA	United States Food and Drug Administration

Abbreviation	Definition
FNA	Fine needle aspiration
GBM	Glioblastoma
GCIG	Gynecologic Cancer Intergroup
GCP	Good Clinical Practice
GLP	Good Laboratory Practice
HBcAb	Hepatitis b core antibody
HBsAg	Hepatitis b surface antigen
HBV	Hepatitis B virus
HCV	Hepatitis C virus
HIPAA	Health Insurance Portability and Accountability Act
HIV	Human immunodeficiency virus
HNSTD	Highest non-severely toxic dose
ICH	International Conference on Harmonization
ICMJE	International Committee of Medical Journal Editors
IHC	Immunohistochemistry
IND	Investigational new drug
INR	International normalized ratio
IP	Intraperitoneal
IRB	Institutional review board
IRR	Infusion-related reaction
IV	Intravenous
LHRH	Luteinizing hormone-releasing hormone
MDSC	Myeloid-derived suppressor cells
MedDRA	Medical Dictionary for Regulatory Activities
µg	Microgram
mg	Milligram
µL	Microliter
mL	Milliliter
mm	Millimeter
MMR	Mismatch repair
mOS	Median overall survival

**Abbreviation**	**Definition**
MPV	Mean platelet volume
MRI	Magnetic resonance imaging
MSI	Microsatellite instable
MSS	Microsatellite stable
MTD	Maximum tolerated dose
NCCN	National Comprehensive Cancer Network
NCI	National Cancer Institute
NOAEL	No-observed-adverse-effect-level
NOEL	No-observed-effect-level
NYHA	New York Heart Association
ORR	Objective response rate
PARP	Poly (ADP-ribose) polymerase
PBMC	Peripheral blood mononuclear cell
PD	Pharmacodynamic(s)
PD-1	Programmed cell death protein 1
PD-L1	Programmed death-ligand 1
PDP	Pharmacodynamic population
PDX	Patient-derived xenograft
PFS	Progression-free survival
PI	Principal investigator
PK	Pharmacokinetic(s)
PKP	Pharmacokinetic population
PP	Per protocol population
PR	Partial response
PS	Performance status
Psap	Prosaposin
PT	Prothrombin time
PTT	Partial thromboplastin time
QD	Once daily
Q3D	Every 3 days
Q7D	Every 7 days

Abbreviation	Definition
RANO	Response Assessment in Neuro-Oncology
RECIST	Response Evaluation Criteria in Solid Tumors
RNA	Ribonucleic acid
RP2D	Recommended Phase 2 dose
SAE	Serious adverse event
SAF	Safety population
SAP	Statistical analysis plan
SD	Stable disease
STD10	Severely toxic in 10% of population
TEAE	Treatment-emergent adverse event
T_{max}	Time to maximum dose concentration
TMB	Tumor mutation burden
TME	Tumor microenvironment
TNBC	Triple negative breast cancer
Tsp-1	Thrombospondin-1
ULN	Upper limit of normal
V_{dss}	Volume of distribution at steady state
WBC	White blood count

2. PROTOCOL SYNOPSIS

**Study Title:** A Phase 1 Study Evaluating the Safety, Pharmacology, and Preliminary Activity of VT1021 in Patients with Advanced Solid Tumors	
**Name of Finished Product:**<br>VT1021	**Name of Active Ingredient:**
**Protocol Number:** VT1021-01	**Study Phase:** 1
**Clinical Sites:**<br>Multiple sites: 3-4 sites in the Dose Escalation Phase; with additional sites as needed to obtain indication-specific patients for each Dose Expansion Phase cohort.	
**Primary Objective:**<br>**Escalation Phase**<br>• To determine the Recommended Phase 2 Dose (RP2D) of VT1021 in patients with advanced solid tumors.<br>**Expansion Phase**<br>• To characterize the safety and tolerability of VT1021 in patient cohorts of specific indications (e.g., ovarian, pancreatic, triple negative breast cancer [TNBC], glioblastoma [GBM], and CD36-high patients).	
**Secondary Objectives:**<br>1. To characterize the adverse event profile of VT1021.<br>2. To determine the pharmacokinetics (PK) of VT1021.<br>3. To describe preliminary evidence of efficacy of VT1021 using objective response rate (ORR), disease control rate (DCR), and progression-free survival (PFS) based on Response Evaluation Criteria in Solid Tumors (RECIST) v1.1 (Appendix 3) or Response Assessment in Neuro-Oncology (RANO) for GBM patients (Appendix 7).<br>4. To determine overall response rate by iRECIST (Appendix 4).	
**Exploratory Objectives:**<br>• To determine the pharmacodynamics (PD) of VT1021.<br>• To assess the effect of VT1021 on thrombospondin-1 (Tsp-1) in circulating peripheral blood mononuclear cells (PBMCs) and plasma.<br>• To assess the effect of VT1021 on circulating levels of immune effector cell populations.<br>• To assess the effect of VT1021 on various tumor microenvironment (TME) characteristics, such as Tsp-1 expression, vascularity and vessel density, and the presence of certain macrophage sub-populations within tumor biopsies.	

Study Design:

This is an open-label Phase I study of VT1021 in patients with advanced solid tumors. Patients must have recurrent or advanced cancer (i.e., solid tumors) for which standard therapy offers no curative potential. The study will include a Dose Escalation Phase and a Dose Expansion Phase. Upon determination of the RP2D in the Dose Escalation Phase, the Dose Expansion Phase will be opened. The Dose Expansion Phase will include cohorts in selected indications (e.g., ovarian, pancreatic, TNBC, GBM, CD36-high), to confirm the safety and tolerability of VT1021 in specific indications.

Patients will receive VT1021 twice per week intravenously (IV) on a 28-day cycle. The extent of disease will be evaluated at the end of Cycle 2 and after every even numbered cycle beyond that (i.e., every 8 weeks). Patients may remain on VT1021 until the occurrence of disease progression or unacceptable toxicity.

<u>Dose Escalation Phase</u>

The dose escalation plan will be a variation to the traditional 3+3 study design. Each cohort will enroll at least 1 patient. If no dose limiting toxicity (DLT) is observed, then the next cohort of patients will be enrolled at the next highest dose level. If one DLT is observed in a cohort of patients, then a minimum of 3 patients will be enrolled at that same dose level. Dose escalation continues until at least 2 patients among a cohort of 6 patients experience DLTs. The RP2D will be defined as the dose level just below the toxic dose level. The total number of patients in the Dose Escalation Phase is expected to be approximately 20 to 30 patients. The total number will depend on the timing and frequency of any dose limiting toxicities.

<u>Dose Expansion Phase</u>

After determination of the RP2D, an expansion phase will be implemented. Five cohorts will be enrolled with approximately16-17 patients will be required to obtain 15 evaluable patients. Patients with ovarian cancer (Cohort A), pancreatic cancer (Cohort B), TNBC (Cohort C), GBM (Cohort D), and a basket cohort of patients expressing high levels of CD36 (Cohort E) will be enrolled. The total number of patients in the Dose Expansion Phase is expected to be approximately 80-85 patients.

Study Population:

<u>Inclusion Criteria</u>

To qualify for enrollment, all the following criteria must be met:

1) Patient must provide written informed consent.
2) Patient is ≥ 18 years of age.
3) **For the Dose Escalation Phase**:

   Patients with advanced solid tumors that are refractory to, or intolerant of, existing therapies known to provide clinical benefit for their condition.

   For the Dose Expansion Phase:

   Patients with advanced solid tumors that are refractory to existing therapies known to provide clinical benefit for their condition. Also, a patient may be intolerant of, not eligible for, or has refused prior standard of care therapies.

   For Cohorts A, B, C, and E, paired tumor tissue will be required for study inclusion. Prior to VT1021 administration, archival tumor tissue obtained ≤ 6 months prior to first

dose of VT1021 or fresh tumor tissue is necessary for testing. An additional tumor biopsy must be obtained after initiation of VT1021 administration (on-study biopsy). For Cohort D (GBM), an on-study biopsy will not be required but tumor tissue must be obtained if the patient undergoes surgery after VT1021 administration has begun.

a) **Cohort A-Ovarian**: Patients with a confirmed diagnosis of unresectable epithelial ovarian, fallopian tube, or primary peritoneal cancer may have received up to 3 prior lines of therapy. BRCA mutant patients are excluded unless they have failed a previous line with a poly (ADP-ribose) polymerase (PARP) inhibitor.

b) **Cohort B-Pancreatic**: Patients with a confirmed diagnosis of pancreatic cancer may have received up to 2 prior lines of therapy.

c) **Cohort C-TNBC**: Patients with a confirmed diagnosis of metastatic TNBC may have received up to 3 prior lines of therapy for metastatic disease.

d) **Cohort D-GBM**: Patients with confirmed relapsed or refractory glioblastoma may have received up to 2 prior lines of systemic therapy including chemotherapy-impregnated wafers applied to the tumor bed.

e) **Cohort E-CD36-high basket cohort**: Patients with solid tumor cancers that have high expression of CD36 by immunohistochemistry (IHC) as determined by a scoring index and that are resistant to or have failed either prior chemotherapy and/or checkpoint inhibitor (including anti-programmed cell death protein-1 [PD-1], anti-programmed death-ligand 1 [PD-L1] and anti-cytotoxic T-lymphocyte associated protein 4 [CTLA-4]). Patients may have received up to 3 prior lines of therapy for metastatic disease.

4) Patient has evaluable or measurable disease by RECIST v1.1 (Appendix 3) or, for patients with GBM, RANO (Appendix 7).

5) Patient has a performance status (PS) of 0-1 on the Eastern Cooperative Oncology Group (ECOG) scale or in the case of GBM patients Karnofsky PS of ≥ 60% (Appendix 2).

6) Patient is at least 21 days removed from therapeutic radiation or chemotherapy prior to the first scheduled day of dosing with VT1021 and has recovered to Grade ≤ 1 (National Cancer Institute [NCI] Common Terminology Criteria for Adverse Events [CTCAE] v5.0, Appendix 5) from all clinically significant toxicities related to prior therapies.

a) For patients receiving nitrosoureas or mitomycin C, the window is 6 weeks.

b) For patients receiving monoclonal antibody therapy, the window is at least one half-life or 4 weeks (whichever is shorter).

7) Patient has adequate organ function defined as:

a) Absolute neutrophil count (ANC) ≥ 1.5 x 10^9/L (1500/µL) and absolute lymphocyte count (ALC) ≥ 7 x 10^9/L (700/µL).

b) Platelet ≥ 100 x 10^9/L.

c) Hemoglobin ≥ 9 g/dL.

d) Activated partial thromboplastin time/ prothrombin time/international normalized ratio (aPTT/PT/INR) ≤ 1.5 x upper limit of normal (ULN) unless the patient is on anticoagulants in which case therapeutically acceptable values (as determined by the investigator) meet eligibility requirements.

  e) Aspartate aminotransferase (AST) or alanine aminotransferase (ALT) ≤ 2.5 × ULN. In the case of known (i.e., radiological or biopsy documented) liver metastasis, serum transaminase levels must be ≤ 5 x ULN.
  f) Total serum bilirubin ≤ 1.5 x ULN (except for patients with known Gilbert's Syndrome ≤ 3 x ULN is permitted).
  g) Renal: Serum creatinine < 2.0 x ULN and creatinine clearance ≥ 50 mL/min/1.73m^2.
  h) Serum albumin > 3 gm/dL.

8) Patient agrees to use acceptable methods of contraception during the study and for at least 90 days after the last dose of VT1021 if sexually active and able to bear or beget children.

Exclusion Criteria

The presence of any of the following will exclude the patient from the study:

1) Diagnosis of another malignancy within the past 2 years (excluding a history of carcinoma in situ of the cervix, superficial non-melanoma skin cancer, or superficial bladder cancer that has been adequately treated, or stage 1 prostate cancer that does not require treatment or requires only treatment with luteinizing hormone-releasing hormone agonists or antagonists if initiated at least 90 days prior to the first dose of VT1021).
2) History of a major surgical procedure or a significant traumatic injury within 14 days prior to commencing study drug, or the anticipation of the need for a major surgical procedure during the course of the study.
3) Treatment with investigational therapy(ies) within 5 half-lives of the investigational therapy prior to the first scheduled day of dosing with VT1021, or 4 weeks if the half-life of the investigational agent is not known, whichever is shorter.
4) Concurrent serious (as determined by the Principal Investigator [PI]) medical conditions, including, but not limited to, New York Heart Association (NYHA) class III or IV congestive heart failure, history of congenital prolonged QT syndrome, uncontrolled infection, active hepatitis B, hepatitis C or human immunodeficiency virus (HIV), or other significant co-morbid conditions that, in the opinion of the Investigator, would impair study participation or cooperation.
5) Pregnant or planning to become pregnant or breast feed while on study.
6) Evidence of symptomatic brain metastases. Patients with treated (surgically excised or irradiated) and stable brain metastases are eligible, assuming the patient has adequately recovered from treatment, the treatment was at least 28 days prior to initiation of study drug, and baseline brain computed tomography (CT) with contrast or magnetic resonance imaging (MRI) within 14 days of initiation of study drug, is negative for new or worsening brain metastases.
7) Other concurrent chemotherapy, immunotherapy, radiotherapy, or investigational anti-cancer therapy.
8) Requirement for palliative radiotherapy to lesions that are defined as target lesions by RECIST/RANO criteria at the time of study entry.
9) Known hypersensitivity to any of the components of VT1021 (sodium, phosphate, dibasic, anhydrous sodium, phosphate, monobasic, monohydrate, mannitol, polysorbate 80) or a severe reaction to PS20- or PS80-containing drugs or investigational agents (e.g.,

amiodarone, Vitamin K, etoposide, docetaxel, cancer vaccine, protein biotherapeutics [like monoclonal antibodies], erythropoietin-stimulating agents, fosaprepitant).<br>10) Chronic, systemically administered glucocorticoids in doses equivalent to >5 mg prednisone daily. Topical, inhalational, ophthalmic, intraarticular, and intranasal glucocorticoids are permitted. Isolated or intermittent use of systemically administered glucocorticoids to treat complications of malignancy, use as a premedication, or as a one-time prep for an imaging procedure is permitted. If patient was on >5 mg prednisone/day equivalent, last dose must have been at least 7 days prior to the first planned dose of study drug. Exception: GBM patients may be on chronically administered glucocorticoids for the control of cerebral edema as long as the dose does not exceed 2 mg dexamethasone/15 mg prednisone/day. For GBM patients requiring larger amounts of glucocorticosteroids, consultation with, and agreement by the medical monitor is required before such patients can be enrolled.<br>11) Patients with active hepatitis B (e.g., hepatitis B surface antigen [HBsAg] reactive) are excluded, however, patients with past hepatitis B virus (HBV) infection or resolved HBV infection (defined as the presence of hepatitis B core antibody [HBcAb] and absence of HBsAg) may be enrolled provided that prior testing/known status for HBV deoxyribonucleic acid (DNA) is negative. Patients with active hepatitis C (e.g., hepatitis C virus [HCV] ribonucleic acid [RNA] [qualitative] are detected) are excluded, however, patients with cured hepatitis C (negative HCV RNA prior test/known status) may be enrolled.
**Test Product, Dose, and Mode of Administration:**<br>VT1021 is provided as a 5 mg/mL solution for IV administration. Dosing will be based on body weight on an mg/kg basis (refer to pharmacy manual for algorithm to calculate volume of study drug (VT1021) and steps for dilution in IV bag). Patients will be dosed twice weekly, and the Investigator may choose either “Monday/Thursday” or “Tuesday/Friday” schedule (±1 day). These identical schedules are provided for scheduling convenience and flexibility.<br>In the Dose Escalation Phase, multiple dose levels will be evaluated sequentially as displayed below. In the event of a de-escalation, the nature of the DLT will be reviewed with medical (i.e., Investigator and Medical Monitor) and statistical input and a recommendation of the appropriate dosing of the next cohort determined. The dose for the Expansion Phase (i.e., RP2D) will be determined in the Dose Escalation Phase.

Dose Level	Twice per week	
	Doses	
	Monday (or Tuesday)	Thursday (or Friday)
-1	0.5 mg/kg	0.5 mg/kg
1	1.0 mg/kg	1.0 mg/kg
2	2.0 mg/kg	2.0 mg/kg
3	3.3 mg/kg	3.3 mg/kg
4	5.1 mg/kg	5.1 mg/kg
5	6.6 mg/kg	6.6 mg/kg
6	8.8 mg/kg	8.8. mg/kg
7	11.8 mg/kg	11.8 mg/kg
8	15.6 mg/kg	15.6 mg/kg
9	20.7 mg/kg	20.7 mg/kg

Reference Product, Dose, and Mode of Administration:

None.

Duration of Treatment:

Patients may remain on VT1021 until disease progression or unacceptable toxicity occurs.

Safety Assessments:

- Adverse events: collected from the time the informed consent is signed through the end of study (EOS) visit.

Pharmacokinetic and Pharmacodynamic Assessments:

- The PK of VT1021 will be determined by measuring plasma VT1021 levels over time, from which various PK parameters will be determined.
- The PD of VT1021 will be assessed by measuring various circulating and tumor-based biomarkers, including Tsp-1 levels in PBMCs and plasma, various sub-populations of circulating immune-based cells and Tsp-1 as well as various other targets of VT1021 (CD36, CD47) on pre- and on-study tumor biopsies in patients whose neoplastic disease is accessible for biopsy (excluding Dose Expansion Cohort D, for which on-study biopsies for GBM are not required).

Preliminary Efficacy Assessments:

- Best overall response will be based on the RECIST 1.1 (Appendix 3) and iRECIST (Appendix 4) criteria or RANO (with iRANO modifications) for GBM patients (Appendix 7) and will represent the best response compared to the extent of disease assessment at Screening.

Statistical Methods:

Study Populations

- Safety Population (SAF): Those patients who receive at least one dose of VT1021 will be included in the safety analysis.

- Per Protocol Population (PP): All patients who receive at least one dose of VT1021 and have at least one on-study tumor assessment scan. The PP patients will be used for the primary analysis for each cohort.
- Evaluable: All patients completing Cycle 1 and receiving at least 75% of the planned doses for the cycle, where missed doses must not have been omitted due to VT1021-related toxicity.
- Pharmacokinetic Population (PKP): SAF patients who have evaluable PK samples.
- Pharmacodynamic Population (PDP): SAF patients that have evaluable PD samples.

General Considerations

Categorical variables will be summarized by frequency distributions (number and percentages of patients) and continuous variables will be summarized by descriptive statistics (sample size, mean, standard deviation, median, minimum, maximum).

Demographics and Baseline Characteristics

The demographic and baseline data will be summarized by dose level and on an indication-specific basis.

Pharmacokinetic Analysis

Serum/plasma samples collected from patients will be analyzed for the concentrations of VT1021 to estimate PK parameters. Standard non-compartmental analysis method will be used to determine the PK parameters of VT1021 including maximum dose concentration (C_{max}), time to maximum dose concentration (T_{max}), area under the curve from time zero to the last measurable concentration (AUC_{0-t}), area under the curve from time zero to infinity ($AUC_{0-\infty}$), clearance, volume of distribution at steady state (V_{dss}), and the terminal elimination half-life. The analyzed PK parameters will be summarized using descriptive statistics, including the median, geometric mean and 95% confidence intervals around parameter estimates by dose level.

Pharmacodynamic Analysis

Tsp-1 levels will be summarized by observed and change from baseline. Change from baseline will be analyzed using a signed rank test. The expression levels of Tsp-1 in circulating PBMCs and plasma and accumulation in the platelets will be used to assess VT1021 activity, and the dose range of VT1021 at which Tsp-1 induction plateaus may be used to identify the RP2D. For the expansion cohorts the ratio of CD8+: FoxP3 positive cells among the CD3+ population will be summarized by observed and change from baseline, when applicable.

All PD parameters will be summarized. When applicable, correlation and testing will be performed in order to evaluate the relationship to outcomes (e.g., ORR). The inference testing will be used in a more descriptive manner in order to plan future studies and understanding of the compound.

Sample Size Determination

No sample size calculations were performed for the dose escalation portion of this study. The Dose Expansion has multiple cohorts to determine proof of concept. Each cohort will be analyzed separately; however, the sample size calculation will be the same across each. All cohorts will be analyzed using the PP.

H_0: ORR ≤ 0.15 versus H_A: ORR > 0.375 will be tested using a 1-sided significance level at 0.10. A total of 15 patients is required to provide 80% power to reject the null hypothesis, if not true. Assuming an attrition rate of 10%, an enrollment of approximately16-17 patients will be required to obtain 15 evaluable patients. Therefore, the number required in total across all five cohorts will be approximately 80-85.

Statistical Analysis

The primary efficacy analysis on each dose expansion cohort will be performed using an exact Clopper-Pearson test on ORR (confirmed complete response [CR] + confirmed partial response [PR]). The estimated rate and exact confidence intervals will be displayed. Disease control rate (DCR) will include patients with confirmed responses and/or stable disease for 4+ months or 2-scans and analyzed using an exact Clopper-Pearson test. The estimated rate and exact confidence intervals will be displayed. PFS will be displayed using Kaplan-Meier estimates.

Safety will be evaluated by various adverse event analysis. The incidence of treatment-emergent adverse events (TEAE), related TEAE, serious TEAE, serious related TEAE, discontinuing due to TEAE, deaths, and others as deemed important will be summarized using frequencies and percentages. Laboratory analytes will be analyzed using shifts from baseline using the CTCAE version 5.0. When appropriate graphical displays will be displayed. Vital signs will be similarly analyzed.

3. INTRODUCTION

3.1. Study Rationale

This study will evaluate the safety, pharmacokinetics (PK), and preliminary clinical activity of a novel anti-cancer drug, VT1021. VT1021 is a 5-amino acid cyclic peptide, the sequence of which was derived from prosaposin (Psap), that stimulates Tsp-1 in the tumor microenvironment (TME). Recent research has shown the importance of the TME in the initiation, progression, and metastasis of neoplastic disease. Various chemokines and cytokines represent the signals that influence the growth of the primary tumor and the spread of metastatic disease to sites that have been prepared to receive the metastases. One of these key mediators of the process of tumor progression is thrombospondin-1 (Tsp-1). Tsp-1 has been shown to induce an anti-angiogenic state and to influence other key processes in neoplastic development such as recognition of cancer cells by tumor associated macrophages. Tsp-1 also has direct tumoricidal effects via binding to CD36 (Dawson et al. 1997, Wang et al. 2016). Tsp-1 production in the TME is in turn under the influence of Psap, a lysosomal co-factor involved in sphingolipid metabolism. VT1021 is a cyclic 5-amino acid peptide from within the active moiety of Psap responsible for Tsp-1 stimulation. VT1021 has been shown to have marked anti-tumor activity (Wang et al. 2016) in multiple in vivo tumor models and was shown to be well tolerated in Good Laboratory Practice (GLP) toxicology testing.

Although there has been recent remarkable progress in the treatment of neoplasia, such as the advent of targeted agents and immune-oncology drugs, metastatic cancer in the adult population continues to have a high degree of morbidity and mortality so that the development of novel agents remains a high unmet medical need. Given this ongoing need and given the activity and animal safety profile of VT1021, risk/benefit considerations favor the initiation of clinical development of VT1021 in adults with advanced malignancies.

Based on the experimental findings, an effort was undertaken to evaluate whether the effect of Psap on Tsp-1 production could be translated into a smaller, more easily deliverable agent than the full Psap molecule. One of the saposin molecules derived from Psap (saposin A) maintained the ability to stimulate Tsp-1 production. Various overlapping 20-amino acid domains within the 81-amino acid saposin A were then examined. Tsp-1 stimulatory activity was found within one of the 20-amino acid domains encompassing amino acids 31-50. Further shortening of the amino acid sequence ultimately resulted in a 5-amino acid peptide (DWLPK) that retained the ability to stimulate Tsp-1 production in $Gr1^+$ cells in mice following administration of conditioned media from a highly metastatic cell line. Further chemistry demonstrated that cyclization of the peptide yielded improved results. VT1021 is the cyclic 5-amino acid peptide DWLPK.

3.2. Thrombospondin-1

Tsp-1 is a large glycoprotein found mostly in the extra-cellular space and over-expressed within the TME by many different tumor types. Although first discovered associated within platelets, subsequent research has shown that other cells are capable of producing Tsp-1, including tumor cells, endothelial cells and immune cells, all components of the TME. Tsp-1 levels have been found to be downregulated in tumors of multiple types of cancer (Bhattacharjee et al. 2001; Chen et al. 2002; Hong et al. 2010; Yoshihara et al. 2009). Moreover, expression of Tsp-1 in the TME is repressed in metastatic tumors as compared to localized primary tumors (Kang et al. 2009).

Tsp-1 inhibits angiogenesis via its interaction with two cell surface receptors, CD47 and CD36. Ligation of Tsp-1 by CD47 results in dissociation of VEGFR2 from CD47, leading to an antiangiogenic state (Kaur et al. 2010). Binding of Tsp-1 to CD36 promotes binding of CD36 to β1 integrin and VEGFR2, resulting in decreased phosphorylation of VEGFR2 and decreasing its angiogenic signal (Zhang et al. 2009). Tsp-1 also has direct tumoricidal effects via its binding to CD36 via induction of apoptosis (Russell et al. 2015). In addition, by binding directly to CD47, Tsp-1 may inhibit the anti-phagocytic role of this surface protein, resulting in enhanced macrophage killing of tumor cells.

There have been several attempts to create Tsp-1 mimetic molecules. These have essentially fallen into 2 classes: those that are targeted to CD47 and those that bind to CD36. Only a few of these have reached the clinic. ABT-510 (ABBOTT) is a peptidomimetic molecule targeted to CD36 that was tested in a Phase 2 study in patients with soft-tissue sarcoma (Baker et al. 2008), and in a second Phase 2 study in patients with renal cell carcinoma (Ebbinghaus et al. 2007). In the sarcoma study, 1 of 20 patients demonstrated an objective response, and the 6-month progression-free survival was 24%. In the renal cell carcinoma study, the objective response rate was 4%. Following these studies, further clinical development of ABT-510 was discontinued. CVX-045 (PFIZER) is another peptidomimetic molecule, which was fused to a proprietary antibody scaffold, which underwent Phase 1 testing in 18 patients with advanced solid malignancies. One patient experienced a response, and there were two serious adverse events (SAEs) attributed to the drug: a case of radiation pneumonitis and a bowel perforation that was fatal (Molckovsky & Siu 2008). Further clinical development of CVX-045 has been discontinued. There are several molecules currently in clinical studies targeting CD47, including a fusion protein (www.ClinicalTrials.gov a) and a monoclonal antibody (www.ClinicalTrials.gov b); results of these studies are pending.

3.3. The Role of Prosaposin in Tsp-1 Production

In order to study the role of Tsp-1 in the TME, a study was performed examining Tsp-1 expression in the microenvironment of tumors formed by two weakly-metastatic cell lines (PC3 and MDA-MB-231), and their highly metastatic derivatives (PC3M-LN4 and MDA-MET). Consistent with the anti-tumorigenic activities of Tsp-1, the tumors formed by the weakly-metastatic cells induced the expression of high levels of Tsp-1 in the TME. Conversely, the tumors formed by the highly metastatic cells virtually silenced the expression of Tsp-1 in the TME. Moreover, expression of Tsp-1 in the TME inversely correlated with the frequency of metastasis. There was a highly statistically significant difference in stromal Tsp-1 levels at the primary tumor site between the animals with the highly-metastatic, versus those with the weakly-metastatic, tumors, suggesting that Tsp-1 levels in the TME of a primary tumor can influence the development of metastases. In addition, mice bearing the PC3 tumors had markedly elevated levels of Tsp-1 in the lung compared to animals bearing the highly metastatic tumors, despite the fact that there were no detectable PC3 cells in the lungs of these mice. This observation indicated that the weakly-metastatic PC3 tumors secreted a factor that could stimulate Tsp-1 both in a paracrine manner in the local TME and a systemic (endocrine-like) manner in distant organs.

In order to identify this Tsp-1 inducing secreted factor, prostatic fibroblasts were cultured with conditioned medium from the weakly-metastatic PC3 cells and Tsp-1 production was found to be increased 4-fold. When the conditioned medium of the PC3 cells was fractionated, using a chromatographic method, and the resultant fractions examined for the ability to stimulate Tsp-1,

two fractions possessed this activity; Psap was present in both active fractions. Psap is a member of the class of sphingolipid activator proteins which are soluble lysosomal cofactors responsible for the metabolism of cellular sphingolipids. Psap is cleaved in the lysosome to produce several smaller molecules termed saposins. Psap can also escape lysosomal capture following synthesis and be excreted into the extracellular compartment (Carvelli et al. 2015).

Examination of the PC3 and PC3M-LN4 cells showed that Psap was expressed at approximately 10-fold higher levels in the weakly-metastatic PC3 cells compared to the highly metastatic PC3M-LN4 cells. Additional experiments demonstrated that Psap stimulates production of Tsp-1 via a p53-mediated mechanism in stromal fibroblasts. Finally, silencing Psap expression in PC3 cells resulted in lower Tsp-1 levels in the TME of the primary tumor, as well as in the lungs, and a concomitant increase in metastatic potential. Conversely, ectopic expression of Psap in the highly metastatic PC3M-LN4 cells resulted in increased Tsp-1 expression in the TME of tumors and a significant decrease in metastatic potential (Kang et al. 2009). The conclusion of the study was that Psap inhibits tumor metastases via both paracrine as well as endocrine (i.e., from a distance) stimulation of stromal Tsp-1 via a p53 dependent pathway (Kang et al. 2009).

In a second study (Catena et al. 2013), conditioned media from weakly metastatic (PC3) and highly metastatic (PC3M-LN4) prostate cancer cell lines were injected into mice and recruitment of CD11b+ cells to the lungs of the treated animals was examined. Both cell lines were capable in inducing the infiltration of CD11b+ cells into the lung, but Tsp-1 levels were upregulated in the lungs of the animals who had received the conditioned medium from the weakly-metastatic cell line; furthermore, the Tsp-1 was primarily confined to the bone marrow-derived CD11b+ cells. In contrast, CD11b+ cells from the bone marrows of the animals treated with the conditioned medium from the highly metastatic cell line contained no Tsp-1. Further analysis of the CD11b+ cells demonstrated that they also carried the Gr1 surface marker, and so had a myeloid ancestry. Finally, when wild-type mice were given a bone marrow transplant with cells from a Tsp-1$^{-/-}$ mice Psap, or a peptide derived from Psap, were unable to inhibit metastasis to the lung (Catena et al. 2013).

Additional experiments in this study demonstrated that when Psap production in the weakly metastatic cell line was silenced by a shRNA, conditioned medium from these cells was unable to increase Tsp-1 production in the lungs of treated animals. The conclusion was that weakly-metastatic tumors expressing Psap elevate Tsp-1 levels in bone marrow derived CD11b+/Gr1+ cells in the pre-neoplastic lungs of mice in an endocrine fashion and that this induction of Tsp-1 mediates the inhibition of metastasis.

3.4. VT1021 Pre-Clinical Pharmacology Studies

VT1021 was evaluated in a variety of tumor xenografts and syngeneic murine tumors for anti-neoplastic activity.

3.4.1. Ovarian Cancer Models

VT1021 was tested in two xenograft models of ovarian cancer utilizing: a human patient-derived xenograft (PDX) ovarian cancer cell metastasis model and a CDX (A2780) ovarian cancer orthotopic primary tumor model in C57 SCID immunocompromised mice. VT1021 was also tested in a syngeneic orthotopic 1D8 model in C57Bl6/J mice (Wang, 2016). All tumor lines

were modified to express luciferase for the purposes of monitoring tumor growth non-invasively, in real time.

In the PDX metastatic model, after 14 days of exposure, the relative luciferase intensity demonstrated an 11-fold reduction and the metastatic lesions in the VT1021-treated mice were ~2.3-fold smaller than those in the saline-treated mice (P =0.046) (Figure 1). Moreover, TUNEL staining revealed that the metastatic lesions in the VT1021-treated mice contained a significantly greater percentage of apoptotic cells compared to control (saline)–treated tumors (59% versus 11.4%) (P < 0.0001). VT1021 was also able to regress primary tumors in both the A2780 xenograft model and the 1D8 syngeneic model.

Figure 1: The effect of VT1021 on the growth of a human ovarian cancer xenograft in SCID mice

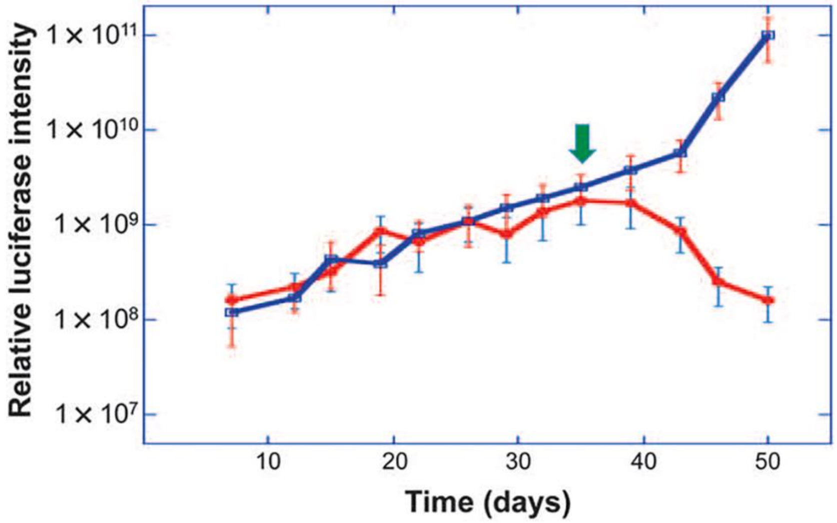

Growth of relative luciferase intensity (which correlates with tumor size) in a PDX model of metastatic ovarian cancer treated with saline (blue line) or VT1021 at 10 mg/kg QD (red line). Green arrow denotes initiation of study drug.

3.4.2. Pancreatic Cancer Model

VT1021 was evaluated in a xenograft model of pancreatic cancer (AsPc1) in C57 SCID mice. SCID mice were injected with $1x10^6$ AsPc1 human pancreatic cancer cells, orthotopically into the head of the pancreas. The animals were treated with vehicle (saline) control or VT1021, once daily (QD) via intraperitoneal (IP) injection for 10 days. The vehicle control group was divided into two cohorts, the first was sacrificed on Day 25 at the initiation of study drug; the second was sacrificed at Day 35 at the endpoint of the experiment. At the experimental endpoint, control treated tumors reached an average mass of 1.6g and had to be euthanized due to morbidity as defined by the institutional animal protocol. Conversely, after only 10 days of study drug, mice treated with VT1021 had an average tumor reduction of 40% from the initiation of study drug, and no detectable metastatic lesions in the liver and spleen (Figure 2).

Figure 2: The effect of VT1021 on the growth of AsPc1 cells in SCID mice

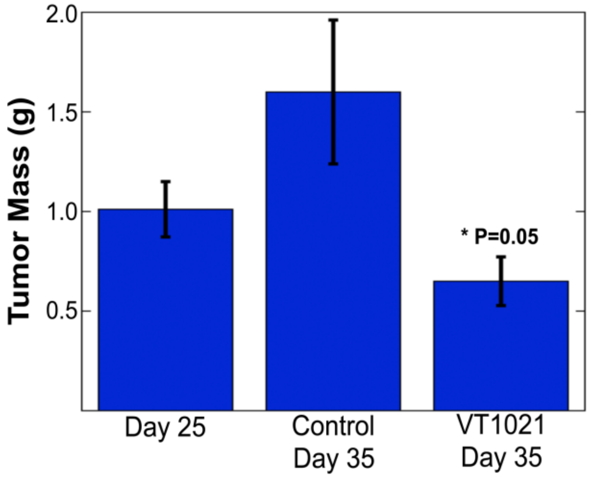

Plot of tumor mass of AsPc1 tumors in SCID mice at Day 25 post-implantation prior to the initiation of study drug and Day 35 post-implantation following 10 days of administration of saline (control) or VT1021 (peptide).

3.4.3. Melanoma Model

VT1021 was evaluated in a murine orthotopic melanoma model against dacarbazine in immunocompetent C57B16/J mice.

Mice were injected with 5x10^5 B16-B16 melanoma cells subcutaneously. Treatment was initiated on Day 8 post-injection when tumors had an average volume of 100 mm^3. Animals were divided into four groups (n=8) and treated with vehicle (saline) control, 40 mg/kg of VT1011 (linear prosaposin peptide), 10 mg/kg of VT1011, or 10 mg/kg of VT1021, administered via IP injection, QD for 15 days.

Mice treated with saline alone had progressive growth of tumor and had to be sacrificed after 21 days. VT1011 at 10 mg/kg inhibited tumor growth, but VT-1021 at 10 mg/kg was as effective as VT1011 at 40 mg/kg (T/C=0.08, P<0.0001) (Figure 3).

Figure 3: Growth of B16 melanoma in syngeneic mice

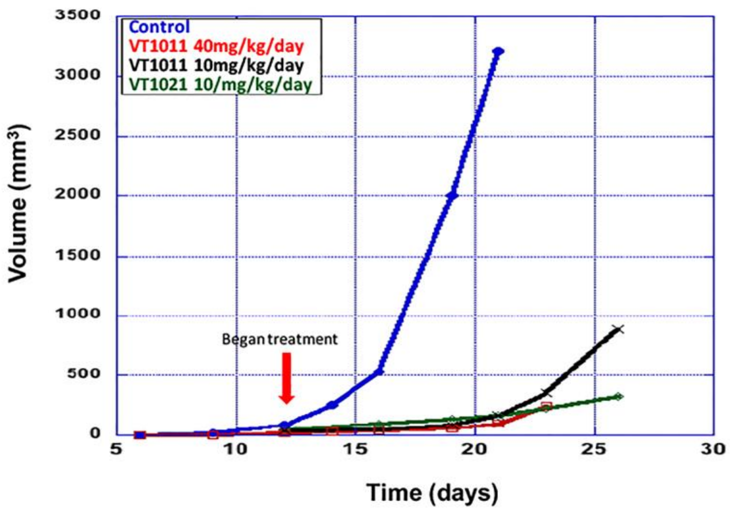

In conclusion, VT1021, when tested against a variety of tumor models, demonstrated promising anti-tumor efficacy.

3.4.4. Triple-Negative Breast Cancer Model

The effect of VT1021 on triple-negative breast cancer (TNBC) was evaluated in athymic nude mice and in a syngeneic model of breast cancer in C57Bl6/J immunocompetent mice.

Athymic nude mice were injected with $1x10^6$ MDA-MB-231 TNBC cells and administered vehicle (saline), 10 mg/kg of VT1021, or 25 mg/kg of VT1021, via IP injection, once daily for 29 days (QDx29). Twelve of 19 mice treated with 10 mg/kg VT1021 and 13 of 19 mice treated with 25 mg/kg VT1021 showed tumor regression or inhibition of tumor growth of 55% or greater, compared to vehicle control, with three tumors regressing to near completion (10 and 25 mg/kg doses; Figure 4 and Figure 5, respectively).

Figure 4: Effect of VT1021 (10 mg/kg; QDx29) on MDA-MB-231 Human TNBC Tumor Growth in Athymic Nude Mice – Individual Animal Findings

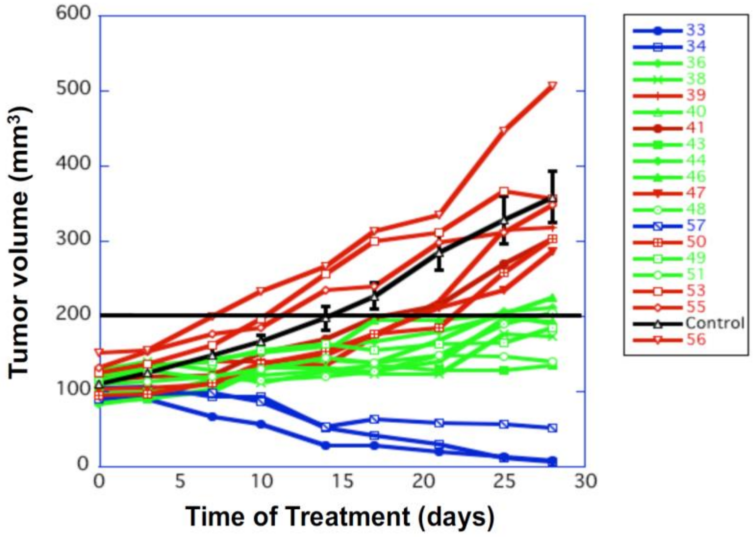

Growth curves of MDA-MB-231 tumors in athymic nude mice treated with saline control (black line) and VT1021 at 10mg/kg; QDx29. Blue lines depict tumors that underwent regression following study drug, green lines represent tumors that grew at a rate <55% of the control, red lines represent tumors whose growth was unaffected by VT1021 compared to control treated tumors.

Figure 5: Effects of VT1021 (25 mg/kg; QDx29) on MDA-MB-231 Human TNBC Tumor Growth in Athymic Nude Mice – Individual Animal Findings

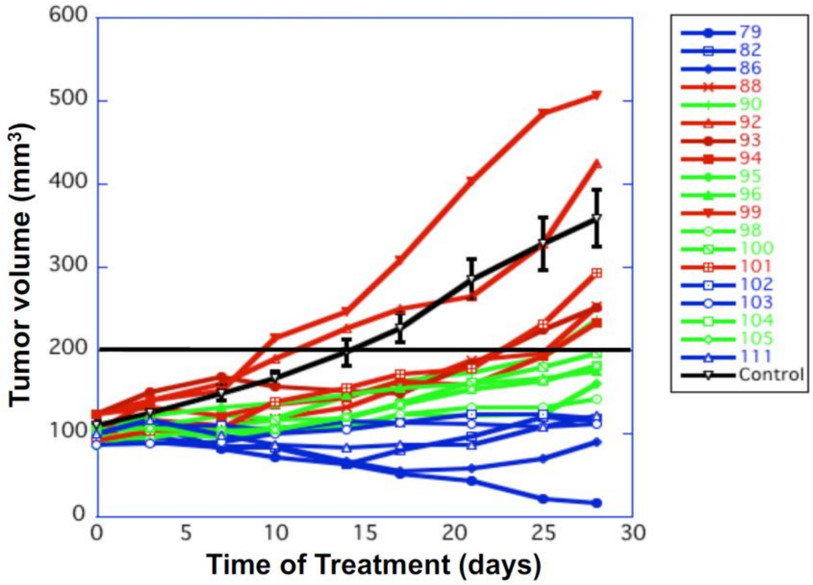

Growth curves of MDA-MB-231 tumors in athymic nude mice treated with saline control (black line) and VT1021 at 25mg/kg; QDx29. Blue lines depict tumors that underwent regression following study drug, green lines represent tumors that grew at a rate <55% of the control, red lines represent tumors whose growth was unaffected by VT1021 compared to control treated tumors.

In a syngeneic model of breast cancer, C57Bl6/J immunocompetent mice were injected with $1x10^6$ E0771 cells. Day 12 post-inoculation, when the average tumor volume for all groups was $75mm^3$, mice were randomized into 4 groups and administered either vehicle (saline) control, VT1021 (10 mg/kg QD), VT1021 (10 mg/kg QD for 5 days, 2 days off, followed by 40 mg/kg every 3 days [Q3D]), or VT1021 (10 mg/kg QD for 5 days, 2 days off, followed by 40 mg/kg every 7 days [Q7D]).

After 26 days of exposure, all of the control-treated mice reached the endpoint criteria of the study and were euthanized. At that time, 62.5% of the mice on the Q3D and Q7D dosing regimens were still alive and 50% of the mice on the QD regimen were still alive (Figure 6). The median overall survival (mOS) for the groups were as follows: QD = 34 days, Q7D = 38 days, Q3D = 45 days. Significantly, on Day 26, when the control mice were euthanized, the average tumor volume was 2,250 mm^3, while the average tumor volumes of the VT1021-treated mice were: QD = 1,050 mm^3, Q7D = 1,050mm^3, and Q3D = 550mm^3 (Figure 7).

Figure 6: Effect of VT1021 on Survival of C57BL6/J Immunocompetent Mice Inoculated with E0771 Breast Cancer Cells

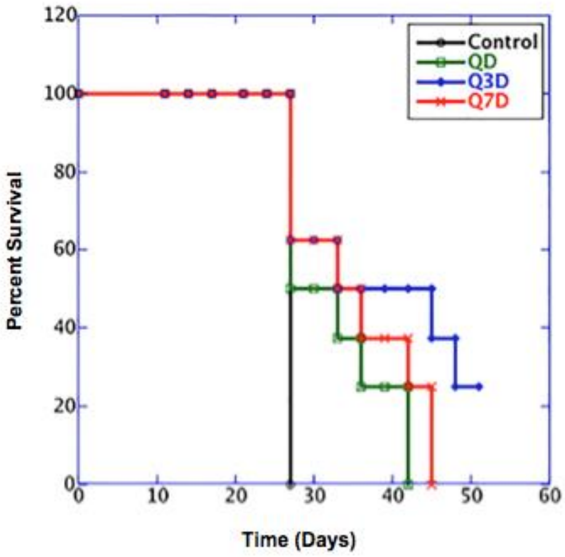

Figure 7: Effect of VT1021 on E0771 Tumor Volume in C57BL6/J Immunocompetent Mice

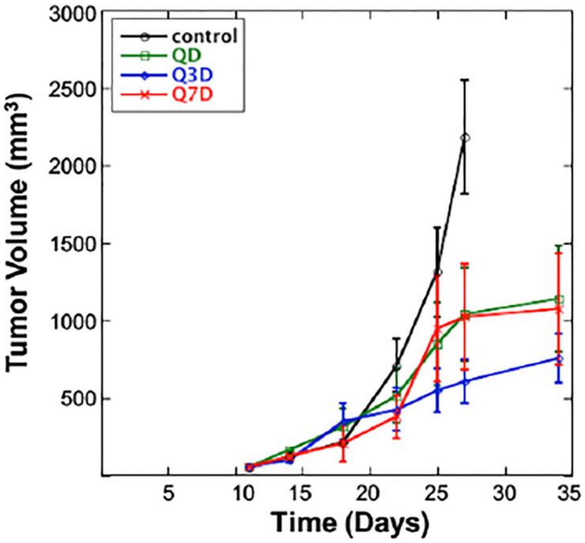

3.5. VT1021 Nonclinical Toxicology

VT1021 was evaluated in GLP-compliant, repeat-dose toxicity studies in rats and in beagle dogs.

In the rat study, animals received vehicle control or VT1021 at 10, 30, or 60 mg/kg, via a 10-minute intravenous (IV) infusion once daily for 28 days. There were no test article-related deaths. There were no test article-related effects on body weight, food consumption, coagulation, serum chemistry, urinalysis, or organ weights during the dosing or recovery periods. There were no test article-related ophthalmic or macroscopic findings during the dosing or recovery periods. The only test article-related microscopic findings after the primary necropsy occurred in the kidney and included degeneration characterized by vacuolation and cellular loss of the renal tubular epithelial cells at the levels of cortico-medullary junction near the arcuate arteries in the 30 mg/kg/day group males and 60 mg/kg/day group males and females. These changes were not considered adverse because of a lack of clinical correlates related to renal clinical pathology parameters, absence of inflammation or overt necrosis, and were absent at recovery. No test article-related microscopic findings were present after the recovery necropsy.

Based on the results of this study, IV administration of VT1021 to Crl:CD(SD) rats at dosage levels of 10, 30, and 60 mg/kg/day for a minimum of 28 days was well tolerated at all dosages. Non-adverse test article-related effects included degeneration of renal tubular epithelium at the level of the arcuate arteries in 30 mg/kg/day group males and in 60 mg/kg/day group males and females. The renal tubular epithelium degeneration was not present after a 28-day recovery period. Therefore, the no-observed-effect level (NOEL) was considered to be 10 mg/kg/day for males and 30 mg/kg/day for females and the no-observed-adverse-effect level (NOAEL) was considered to be 60 mg/kg/day for males and females.

In the dog study, animals received vehicle control or VT1021 at 5, 15 or 35 mg/kg, via a 10-minute IV infusion once daily for 28 days. There were no test-article related effects on body weight, food consumption, coagulation, serum chemistry, urinalysis, or organ weights during the

dosing or recovery periods. There were also no test article-related ophthalmic findings and electrocardiograms were normal in all test article-treated dogs. There were no test article-related clinical observations noted during the recovery period. Based on these results, VT1021 was well tolerated at all administered doses. The NOEL was 5 mg/kg/day, and the NOAEL was 35 mg/kg/day for males and females.

3.6. Rationale for Human Starting Dose and Dose Escalation Schedule

Starting doses for the clinical study were calculated as specified in S9 Nonclinical Evaluation guideline as 1/10 the dose that is observed to be severely toxic in 10% of rodents (STD 10); if the nonrodent is the most appropriate species, then 1/6 the highest non-severely toxic dose (HNSTD) is considered an appropriate starting dose (ICH S9 Guidance for Industry; Nonclinical Evaluation for Anticancer Pharmaceuticals, 2010). For VT1021, a severely toxic dose was not identified in the 28-day, repeat-dose GLP toxicity studies in either rats or dogs. A human starting dose recommendation was therefore made based on the highest doses tested in the rat and dog toxicity studies, using the STD 10/HNSTD approaches typically employed for small molecules. The 60 mg/kg high dose in rats translates, via a body surface area conversion, to 9.7 mg/kg in humans, 1/10 of which is 0.97 mg/kg. The 35 mg/kg high dose in dogs translates similarly to 19.4 mg/kg in humans, 1/6 of which is 3.2 mg/kg. While these recommendations reflect conservative projections, a human starting dose of 1 mg/kg is recommended. Of note, the proposed starting dose in patients is within the range of projected therapeutic efficacious dose range, based on data obtained from various preclinical tumor models, in which efficacy has been observed between 10 and 40 mg/kg, which translates, via a body surface area conversion, to between 0.8 and 3.3 mg/kg in humans.

The dosing schedule being employed is twice weekly IV administration. Based on pre-clinical pharmacology data, it is expected that two doses per week administration is sufficient to induce a pharmacodynamic (PD) response at an efficacious dose. The chosen schedule is designed to provide an opportunity to assess safety and tolerability under a clinically feasible regimen at each escalation level and to evaluate the patients’ responses.

As the study progresses to the highest dose levels, the extent, accumulation, and durability of the responses under this dosing paradigm will be analyzed within the same individuals. This process will allow the assessment of the proposed dosing schedule to determine whether less frequent dosing (i.e., weekly) would provoke a comparable or better effect. PK/PD modelling will be employed as appropriate to determine the optimal dosing schedule for the Dose Expansion Phase and for subsequent studies.

Dose escalation decisions will be predicated on the design described in Section 5.1. Whether to dose escalate beyond the highest listed in the escalation table (20.7 mg/kg) will be determined based on safety, tolerability, response, and PK/PD assessments.

4. OBJECTIVES

4.1. Primary Objective – Dose Escalation Phase

To determine the Recommended Phase 2 Dose (RP2D) of VT1021 in patients with advanced solid tumors.

4.2. Primary Objective – Dose Expansion Phase

To characterize the safety and tolerability of VT1021 in patient cohorts of specific indications (e.g., ovarian, pancreatic, TNBC, glioblastoma [GBM], and CD36-high patients).

4.3. Secondary Objectives

- To characterize the adverse event (AE) profile.
- To determine the pharmacokinetics of VT1021.
- To describe preliminary evidence of efficacy using objective response rate (ORR), disease control rate (DCR), and progression-free survival (PFS) based on RECIST v1.1 (Appendix 3) or Response Assessment in Neuro-Oncology (RANO) with iRANO modifications for GBM patients (Appendix 7).
- To determine overall response rate by iRECIST (Appendix 4).

4.4. Exploratory Objectives

- To determine the pharmacodynamics (PD) of VT1021.
  - To assess the effect of VT1021 on thrombospondin-1 (Tsp-1) in circulating peripheral blood mononuclear cells (PBMCs) and plasma.
  - To assess the effect of VT1021 on circulating levels of immune effector cell populations.
  - To assess the effect of VT1021 on various tumor microenvironment (TME) characteristics, such as Tsp-1 expression, vascularity and vessel density, and the presence of certain macrophage sub-populations with tumor biopsies.

5. STUDY DESIGN

This is an open-label Phase I study of VT1021 in patients with advanced solid tumors. The study will include Dose Escalation and Dose Expansion Phases. Patients must have recurrent or advanced cancer (i.e., solid tumors) for which standard therapy offers no curative potential.

Patients receive VT1021 twice weekly (i.e., M/Th or Tu/Fr) IV on a 28-day cycle. The extent of disease will be evaluated at the end of Cycle 2 and after every even numbered cycle beyond that (i.e., every 8 weeks).

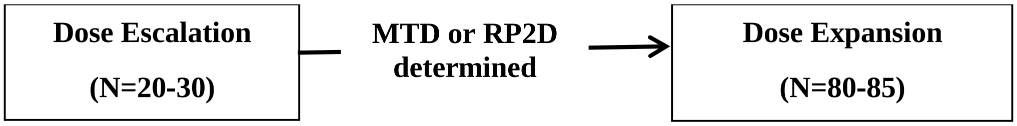

5.1. Dose Escalation Phase

The dose escalation plan will be a variation to the traditional 3+3 study design. Each cohort will enroll at least 1 patient. If no dose-limiting toxicity (DLT) is observed, then the next cohort of patients will be enrolled at the next highest dose level. If one DLT is observed in a cohort of 3 patients, then a minimum of 3 additional patients will be enrolled at that same dose level. Dose escalation continues until at least 2 patients in a cohort of 6 patients experience DLTs. The recommended dose for the study will be defined as the dose level just below the toxic dose level. The total number of patients in the Dose Escalation Phase is expected to be approximately 20 and 30 patients. The total number will depend on the timing and frequency of any DLTs.

The dose escalation component of this Phase 1 study will commence with accrual of at least one patient to Dose Level 1 (i.e., 1 mg/kg administered on Monday/Thursday or Tuesday/Friday). In the event of DLT at the starting dose (1 mg/kg), a lower dose of 0.5 mg/kg (Dose Level -1) will be investigated. To accommodate logistics of enrollment at multiple centers, over-enrollment of a cohort is allowed. If more than one patient is enrolled into any cohort, the patients may be enrolled concurrently. All patients enrolled in any cohort must complete Cycle 1 DLT evaluation before dosing is initiated at the next higher level. See Section 7.5 for Dose Level details.

In the absence of Grade 2 (or greater) AE occurring during Cycle 1, subsequent cohorts will continue by enrolling, at minimum, one patient per cohort. Following the occurrence of any Grade 2 (or greater) AE occurring during Cycle 1 for which there is no clear alternative explanation for causality other than attribution to VT1021, the escalation design will change to (at minimum) three patients per cohort and follow the dose escalation rules that follow:

- If 0 of 3 or ≤1 of 6 patients experience a DLT, the dose level may be escalated.
- If 1 of 3 patients experience a DLT, another 3 patients will be evaluated at the current dose level.
- If ≥ 2 of 3 or ≥ 2 of 6 patients experience a DLT at any dose level, the Dose Escalation Phase of the study will be completed, and the dose immediately below the current level will be the maximum tolerated dose (MTD) (assuming that no more than 1 of 6 patients experienced a DLT at the MTD level).

A DLT will be defined as one or more specified toxicities occurring in Cycle 1 during dose escalation. See Section 9.1.5 for details. Only toxicities occurring during Cycle 1 of study therapy will be considered as DLTs and utilized to inform dose escalation decisions. As safety data become available for patients remaining on-study after Cycle 1, these data will be taken into consideration by the Sponsor when making decisions about continued dose-escalation and defining a RP2D. Enrollment in the Dose Expansion Phase of the study may commence once the RP2D has been determined.

Patients will be treated in repeated 28-day cycles until progression or another withdrawal criterion is met.

Intra-patient dose escalation:

Intra-patient dose escalation should be considered if the following conditions have been met:

- The patient has completed at least two cycles of study drug at the current dose level with no VT1021-related, clinically meaningful Grade 3 or Grade 4 AEs
- No fewer than 3 patients have been treated at the next dose level, and have completed Cycle 1 with no VT1021-related clinically meaningful Grade 3 or Grade 4 AEs

If any significant safety issues occur following the intra-patient dose escalation, the patient is allowed to return to originally administered dose level.

The study Schedule of Assessments and Procedures can be found in Appendix 1.

5.2. Dose Expansion Phase

After determination of the RP2D is completed, the Dose Expansion Phase will be implemented. Cohorts will be enrolled with approximately 16-17 patients to obtain 15 evaluable patients. Patients with ovarian cancer (Cohort A), pancreatic cancer (Cohort B), TNBC (Cohort C), GBM (Cohort D), and a basket cohort of patients with solid tumor cancers expressing high levels of CD36 (Cohort E) will be enrolled. In Cohort D, prior to VT1021 administration, archival tumor tissue obtained ≤ 6 months prior to first dose of VT1021 or fresh tumor tissue is necessary for testing. Patients treated with the RP2D during the Dose Escalation Phase may be included as part of the Expansion cohort of patients per indication. The total number of patients in the Dose Expansion Phase is expected to be approximately 80-85 patients.

Dosing in the Expansion Phase will continue until criteria for study drug discontinuation are met (disease progression, toxicity, or withdrawal from the study). Toxicities during the Dose Expansion Phase are to be monitored according to the Toxicity Management Plan.

The study Schedule of Assessments and Procedures can be found in Appendix 1.

5.3. Determination of Maximum Tolerated Dose and Recommended Phase 2 Dose

5.3.1. Maximum Tolerated Dose

The maximum-tolerated dose (MTD) is defined as one dose level below the dose in which DLTs are observed in >33% of patients.

5.3.2. Recommended Phase 2 Dose

The RP2D will be determined by assessing the overall safety and tolerability, PK, PD and preliminary clinical activity and the RP2D may be equal to or lower than the MTD.

6. ENROLLMENT

6.1. Sample Size

It is anticipated that the Dose Escalation Phase of the study will be completed upon enrollment of approximately 20 to 30 patients. A lower number of patients will be enrolled if all dose levels are cycled without an experience of a DLT or if the PD analyses indicate, for example, maximum induction of Tsp-1 levels.

Once the RP2D has been determined, Dose Expansion cohorts will be initiated with dosing of VT1021 at the RP2D. Each Expansion cohort will enroll no fewer than 6 patients in order to establish a "safety cohort" for that indication. It is anticipated that the Dose Expansion Phase of the study will be completed upon enrollment of approximately 80-85 patients.

6.2. Study Sites

Patients will be enrolled from 2-3 investigational sites during the Dose Escalation Phase of the study with additional sites included to obtain indication-specific patients for each Dose Expansion Phase cohort.

6.3. Inclusion Criteria

To qualify for enrollment, all the following criteria must be met:

1) Patient must provide written informed consent.
2) Patient is ≥ 18 years of age.
3) **For the Dose Escalation Phase**:

   Patients with advanced solid tumors that are refractory to, or intolerant of, existing therapies known to provide clinical benefit for their condition.

   For the Dose Expansion Phase:

   Patients with advanced solid tumors that are refractory to existing therapies known to provide clinical benefit for their condition. Also, a patient may be intolerant of, not eligible for, or has refused prior standard of care therapies.

   For Cohorts A, B, C, and E, paired tumor tissue will be required for study inclusion. Prior to VT1021 administration, archival tumor tissue obtained ≤ 6 months prior to first dose of VT1021 or fresh tumor tissue is necessary for testing. An additional tumor biopsy must be obtained after initiation of VT1021 administration (on-study biopsy). For Cohort D (GBM), an on-study biopsy will not be required but tumor tissue must be obtained if the patient undergoes surgery after VT1021 administration has begun.

   a) **Cohort A-Ovarian**: Patients with a confirmed diagnosis of unresectable epithelial ovarian, fallopian tube, or primary peritoneal cancer may have received up to 3 prior lines of therapy. BRCA mutant patients are excluded unless they have failed a previous line with a poly (ADP-ribose) polymerase (PARP) inhibitor.
   b) **Cohort B-Pancreatic**: Patients with a confirmed diagnosis of pancreatic cancer may have received up to 2 prior lines of therapy.

c) **Cohort C-TNBC**: Patients with a confirmed diagnosis of metastatic TNBC may have received up to 3 prior lines of therapy for metastatic disease.

d) **Cohort D-GBM**: Patients with confirmed relapsed or refractory glioblastoma may have received up to 2 prior lines of systemic therapy including chemotherapy-impregnated wafers applied to the tumor bed.

e) **Cohort E-CD36-high basket cohort**: Patients with solid tumor cancers that have high expression of CD36 by immunohistochemistry (IHC) as determined by a scoring index and that are resistant to or have failed either prior chemotherapy and/or checkpoint inhibitor (including anti-programmed cell death protein-1 [PD-1], anti-programmed death-ligand 1 [PD-L1] and anti-cytotoxic T-lymphocyte associated protein 4 [CTLA-4]). Patients may have received up to 3 prior lines of therapy for metastatic disease.

4) Patient has evaluable or measurable disease by RECIST v1.1 (Appendix 3) or, for patients with GBM, RANO (Appendix 7).

5) Patient has a performance status (PS) of 0-1 on the Eastern Cooperative Oncology Group (ECOG) scale or in the case of GBM patients Karnofsky PS of ≥ 60% (Appendix 2).

6) Patient is at least 21 days removed from therapeutic radiation or chemotherapy prior to the first scheduled day of dosing with VT1021 and has recovered to Grade ≤ 1 (National Cancer Institute [NCI] Common Terminology Criteria for Adverse Events [CTCAE] v5.0, Appendix 5) from all clinically significant toxicities related to prior therapies.

   a) For patients receiving nitrosoureas or mitomycin C, the window is 6 weeks.

   b) For patients receiving monoclonal antibody therapy, the window is at least one half-life or 4 weeks (whichever is shorter).

7) Patient has adequate organ function defined as:

   a) Absolute neutrophil count (ANC) ≥ 1.5 x 10^9/L (1500/µL) and absolute lymphocyte count (ALC) ≥ 7 x 10^9/L (700/µL).

   b) Platelet ≥ 100 x 10^9/L.

   c) Hemoglobin ≥ 9 g/dL.

   d) Activated partial thromboplastin time/ prothrombin time/international normalized ratio (aPTT/PT/INR) ≤ 1.5 x upper limit of normal (ULN) unless the patient is on anticoagulants in which case therapeutically acceptable values (as determined by the investigator) meet eligibility requirements.

   e) Aspartate aminotransferase (AST) or alanine aminotransferase (ALT) ≤ 2.5 × ULN. In the case of known (i.e., radiological or biopsy documented) liver metastasis, serum transaminase levels must be ≤ 5 x ULN.

   f) Total serum bilirubin ≤ 1.5 x ULN (except for patients with known Gilbert's Syndrome ≤ 3 x ULN is permitted).

   g) Renal: Serum creatinine < 2.0 x ULN and creatinine clearance ≥ 50 mL/min/1.73m^2.

   h) Serum albumin > 3 gm/dL.

8) Patient agrees to use acceptable methods of contraception during the study and for at least 90 days after the last dose of VT1021 if sexually active and able to bear or beget children.

6.4. Exclusion Criteria

The presence of any of the following will exclude the patient from the study:

1) Diagnosis of another malignancy within the past 2 years (excluding a history of carcinoma in situ of the cervix, superficial non-melanoma skin cancer, or superficial bladder cancer that has been adequately treated, or stage 1 prostate cancer that does not require treatment or requires only treatment with luteinizing hormone-releasing hormone agonists or antagonists if initiated at least 90 days prior to the first dose of VT1021).
2) History of a major surgical procedure or a significant traumatic injury within 14 days prior to commencing study drug, or the anticipation of the need for a major surgical procedure during the course of the study.
3) Treatment with investigational therapy(ies) within 5 half-lives of the investigational therapy prior to the first scheduled day of dosing with VT1021, or 4 weeks if the half-life of the investigational agent is not known, whichever is shorter.
4) Concurrent serious (as determined by the Principal Investigator [PI]) medical conditions, including, but not limited to, New York Heart Association (NYHA) class III or IV congestive heart failure, history of congenital prolonged QT syndrome, uncontrolled infection, active hepatitis B, hepatitis C or human immunodeficiency virus (HIV), or other significant co-morbid conditions that, in the opinion of the Investigator, would impair study participation or cooperation.
5) Pregnant or planning to become pregnant or breast feed while on study.
6) Evidence of symptomatic brain metastases. Patients with treated (surgically excised or irradiated) and stable brain metastases are eligible, assuming the patient has adequately recovered from treatment, the treatment was at least 28 days prior to initiation of study drug, and baseline brain computed tomography (CT) with contrast or magnetic resonance imaging (MRI) within 14 days of initiation of study drug, is negative for new or worsening brain metastases.
7) Other concurrent chemotherapy, immunotherapy, radiotherapy, or investigational anti-cancer therapy.
8) Requirement for palliative radiotherapy to lesions that are defined as target lesions by RECIST/RANO criteria at the time of study entry.
9) Known hypersensitivity to any of the components of VT1021 (sodium, phosphate, dibasic, anhydrous sodium, phosphate, monobasic, monohydrate, mannitol, polysorbate 80) or a severe reaction to PS20- or PS80-containing drugs or investigational agents (e.g., amiodarone, Vitamin K, etoposide, docetaxel, cancer vaccine, protein biotherapeutics [like monoclonal antibodies], erythropoietin-stimulating agents, fosaprepitant).
10) Chronic, systemically administered glucocorticoids in doses equivalent to >5 mg prednisone daily. Topical, inhalational, ophthalmic, intraarticular, and intranasal glucocorticoids are permitted. Isolated or intermittent use of systemically administered

glucocorticoids to treat complications of malignancy, use as a premedication, or as a one-time prep for an imaging procedure is permitted. If patient was on >5 mg prednisone/day equivalent, last dose must have been at least 7 days prior to the first planned dose of study drug. Exception: GBM patients may be on chronically administered glucocorticoids for the control of cerebral edema as long as the dose does not exceed 2 mg dexamethasone/15 mg prednisone/day. For GBM patients requiring larger amounts of glucocorticosteroids, consultation with and agreement by the medical monitor is required before such patients can be enrolled.

11) Patients with active hepatitis B (e.g., hepatitis B surface antigen [HBsAg] reactive) are excluded, however, patients with past hepatitis B virus (HBV) infection or resolved HBV infection (defined as the presence of hepatitis B core antibody [HBcAb] and absence of HBsAg) may be enrolled provided that prior testing/known status for HBV deoxyribonucleic acid (DNA) is negative. Patients with active hepatitis C (e.g., hepatitis C virus [HCV] ribonucleic acid [RNA] [qualitative] are detected) are excluded, however, patients with cured hepatitis C (negative HCV RNA prior test/known status) may be enrolled.

6.5. Patient Discontinuation

Patients may withdraw from the study at any time and for any reason. If a patient withdraws from the study, appropriate attempts should be made to contact the patient to determine the reason(s) for discontinuation. A complete final evaluation at the time of the patient’s withdrawal should be made with an explanation of why the patient is withdrawing from the study.

Some possible reasons for early withdrawal from the study or study drug include, but are not limited to the following:

- Progressive neoplastic disease
- Symptomatic deterioration
- AE including DLT
- Patient withdraws consent to continue participation
- Study termination by the Sponsor
- Patient is lost to follow up
- Protocol violation/noncompliance
- Death
- Other reason

Patients should be evaluated as soon as possible after ending study drug. All patients will enter a follow-up period of 30 days and be assessed again to assure patient safety. All procedures and evaluations required by the 30-day follow-up visit should also be completed when a patient discontinues study drug. If a patient does not return to the clinic for the overall survival follow-up visit, at least 3 documented attempts should be made to contact the patient. The date of death may be captured from public records.

All patients who discontinue study drug as a result of an AE must be followed until resolution or stabilization of the AE.

6.6. Patient Replacement

Patients who discontinue the study within the first 4 weeks for reasons other than DLT will be replaced. No other patient replacement will take place during the study.

7. VT1021 TREATMENT

All study drug packaging and labeling will be in accordance with applicable local regulatory requirements and applicable Good Manufacturing Practice guidelines.

7.1. Administration and Description

VT1021 is a cyclic pentapeptide containing all L-amino acids. VT1021, as the acetate salt, is formulated with phosphate-buffered saline, mannitol, and 1% Polysorbate 80 and provided as a sterile solution, 5 mg/mL strength, in 10 mL glass vial closed with an elastomeric stopper and crimp seal. Each vial contains 10 mL for a total of 50 mg of VT1021 per vial.

Study drug will be administered in a dose calculated according to a patient's body weight obtained at Baseline. This dose will remain the same unless there has been a ≥10% change in body weight since baseline or as per institutional standard, in which case the current weight will be used for dosing in that cycle. The calculation of the patient's dose is to be documented in the eCRF.

For Dose Levels 1-4, the appropriate volume of VT1021 will be removed from the vial(s) and transferred into a 250 mL bag of either 0.9% saline (normal saline) or D5W and administered IV for no less than 30 minutes. For Dose Level 5 and above, the appropriate volume of VT1021 will be removed from vials and transferred to a 500 mL IV bag and administered IV for no less than 60 minutes. A volume of NS or D5W equivalent to that of VT1021 must be removed from the bag to ensure the total volume remains 500 mL. VT1021 may be administered via a peripheral IV line, or directly into an indwelling IV access device such as a port or a peripherally inserted central catheter ("PICC line"). Once diluted and removed from refrigeration, VT1021 should be administered within 4 hours. (Refer to pharmacy manual for algorithm to calculate volume of VT1021).

7.1.1. Premedication Regimen

Prior to receiving each infusion of VT1021 during the first week of the first cycle of therapy, patients are required to receive premedication with:

- Dexamethasone 10 mg by mouth (PO) approximately 6-12 hours pre-infusion. Alternatively, glucocorticoid therapy equivalent to 80-100 mg IV methylprednisolone may be administered 0.5-2 hours prior to the start of infusion.
- Antihistamine (H1 antagonist) equivalent to 25-50 mg IV/PO diphenhydramine approximately 0.5 hours prior to the start of infusion.
- Acetaminophen 650 mg PO approximately 0.5 hours prior to the start of infusion.
- H2 blockers (e.g., ranitidine) can be used at the discretion of the Investigator.

Note that, in lieu of the regimen listed above, Clinical Investigators may administer pre-medication regimens as per their institutional guidelines.

- For patients that experience mild to moderate infusion reactions, additional glucocorticoid and anti-histamine therapy may be administered as needed and with the resolution of symptoms, the infusion may be resumed at 1/2 half rate at the Investigator's discretion.

- In patients that tolerate the VT1021 infusion during the first week of the first cycle of administration, the premedication corticosteroid dose can be decreased, tapered, or eliminated according to Investigator discretion.

7.2. Storage, Handling, Packaging, and Labeling

Vials must be stored refrigerated (2° - 8°C). Vials will be packaged to a labeled, plain, white cardboard carton, with 25 vials per carton. Vials must be protected from light until immediately before use. VT1021 does not require re-constitution and does not require agitation prior to use. The label specifies the amount of drug delivered when removed with a syringe, and indicates the drug is for investigational use only.

7.3. Duration of Study Drug

Patients may remain on VT1021 until disease progression, or unacceptable toxicity.

7.4. Dosing Schedules

7.4.1. Dose Escalation Phase

VT1021 will be administered as a twice per week IV infusion. Dose escalation/de-escalation levels will be determined based on the timing and severity of any DLTs. If, in the Investigator's judgement, a patient is not tolerating a dose level, the Medical Monitor should be consulted regarding dose reduction. See Section 7.5 for Dose Levels.

7.4.2. Dose Expansion Phase

Patients will be dosed twice per week at the R2PD as determined by the Dose Escalation Phase. If possible, toxicities are to be managed symptomatically. The appropriate treatment should be used to ameliorate symptoms. After discussion with the Medical Monitor, dose reductions are allowed for drug-related toxicities; however, no more than 2 dose reductions are allowed for each patient, with no dose re-escalation.

7.5. Dose Escalation/De-escalation Levels

Table 1 details the Dose Levels to be used in the Dose Escalation Phase. See Section 5.1 for dose escalation and de-escalation rules.

Table 1: VT1021 Dose Levels

Dose Level	Twice per week	
	Doses	
	Monday (or Tuesday)	Thursday (or Friday)
-1	0.5 mg/kg	0.5 mg/kg
1	1.0 mg/kg	1.0 mg/kg
2	2.0 mg/kg	2.0 mg/kg
3	3.3 mg/kg	3.3 mg/kg
4	5.1 mg/kg	5.1 mg/kg
5	6.6 mg/kg	6.6 mg/kg
6	8.8 mg/kg	8.8. mg/kg
7	11.8 mg/kg	11.8 mg/kg
8	15.6 mg/kg	15.6 mg/kg
9	20.7 mg/kg	20.7 mg/kg

7.6. Re-initiation of VT1021 after Dose Limiting Toxicity

Patients who experience a DLT may not receive additional doses of VT1021 at the same dose level. However, if there is evidence that a patient who experiences a DLT has also derived clinical benefit from VT1021, then the Investigators, Medical Monitor and Sponsor will review the specifics of the case. Such a patient could continue on study at a lower dose level if the consensus judgment is that continued VT1021 is in the patient's best interest. All non-hematologic/metabolic AEs should have recovered to CTCAE v5.0 Grade 0, Grade 1, or baseline (except alopecia) prior to the administration of additional cycles of VT1021.

7.7. Guidelines for Dose Modification

Patients who experience a DLT determined to be from the dosing of VT1021 will have study drug suspended until resolution of the DLT to Grade ≤ 1 or to a pre-study degree of severity. The patient may then begin dosing at one dose level below the current dosing level. Patients in whom a DLT does not reverse to Grade ≤ 1 within 2 weeks will be discontinued from the study.

Patients who experience a toxicity described under the definition of a DLT following dose reduction will be discontinued from the study.

Discontinuation of VT1021 for individual patients should be considered in the case of any of the following:

- ALT or AST >8×ULN.
- ALT or AST >5×ULN for more than 2 weeks.
- ALT or AST >3×ULN **and** (total bilirubin >2×ULN or INR >1.5).

- ALT or AST >3×ULN with the appearance of fatigue, nausea, vomiting, right upper quadrant pain or tenderness, fever, rash, and/or eosinophilia (>5%).

Patients experiencing adverse events due to VT1021 that do not meet the formal definition of a DLT and who require the dosing to be discontinued may be restarted at a lower dose after the adverse events are Grade ≤1 and a discussion has occurred with accord from the Medical Monitor.

7.8. Management of Infusion-related Reactions

7.8.1. Reflex Testing for Infusion-related Reactions

Patients are to be closely monitored for infusion-related reactions (IRR) throughout study drug administration including during and after VT1021 administration. Signs and symptoms of IRR may vary among patients, and may include, but are not limited to:

- Fever.
- Chills, rigors, myalgia.
- Facial or systemic erythema, pallor, facial swelling.
- Chest tightness, difficulty breathing, wheezing, stridor, tachypnea, bronchospasm, cough.
- Tachycardia or significant pulse rate increase from baseline without other obvious cause.
- Bradycardia or significant pulse rate decrease from baseline.
- Pre-syncope or syncope.
- Hypotension, orthostatic hypotension, or blood pressure swings, including hypertension.
- Skin rash, urticaria, angioedema, or pruritus.
- Swelling of the throat, tongue, mouth, or lip.
- Difficulty speaking; hoarse or raspy voice.
- Excessive salivation; difficulty swallowing.
- Nausea, vomiting, cramps, or diarrhea.
- Development of a headache, especially moderate or greater, after start of infusion.

An IRR could be occurring when any symptoms suggestive of such a reaction begin during the study drug infusion, after infusion during the observation period, or days to weeks later. While these symptoms may be self-limiting, such symptoms may signal the possibility of a severe reaction that could escalate into a life-threatening situation.

In the event of a ≥Grade 2 IRR, study drug infusion must be stopped immediately and appropriate medical care must be given. If necessary, the patient should be transferred to an acute care facility.

After the patient is stabilized, additional blood samples are to be collected and procedures performed, depending on the IRR, to include but not be limited to:

- Hematology
- Clinical chemistry
- Complement panel (CH50, C3a, C5a)
- Tryptase
- Plasma VT1021 levels
- Inflammatory cytokines (IL-1, IL-2, IL-6, IL-12, IL-18, tumor necrosis factor-α, interferon [IFN]-γ, and GM-CSF).

The Investigator must inform the study Medical Monitor promptly regarding any patient who experiences a ≥Grade 2 IRR for additional instruction.

Patients who experience ≥Grade 2 IRR will continue to be monitored / have repeat assessments performed at a schedule determined by the Investigator/institutional guidelines in consultation with the Medical Monitor and Sponsor. Appropriate follow up for the patient must occur to ensure that there are no late-occurring sequelae.

7.8.2. Infusion-related Reactions Related to VT1021

If a patient has a Grade 1 infusion reaction with the first infusion of VT1021, the infusion must be slowed immediately. If symptoms persist or worsen to ≥Grade 2, consideration must be given to medical therapy, including epinephrine (e.g., epinephrine autoinjector such as the EpiPen™), IV corticosteroids, IV antihistamines, bronchodilators, oxygen, and vasopressors, and other supportive measures per institutional guidelines, based on the clinical manifestations and severity grade.

For patients who experience ≥Grade 3 infusion reactions with the first VT1021 infusion, re-challenge must not be done and study drug must be discontinued.

For ≤Grade 2 infusion reactions, subsequent infusions may be given at the Investigator's discretion. However, for subsequent infusions, the pre-medications listed in Section 7.1.1 must be administered and the infusion should be at a slower rate (e.g., over 90 minutes rather than the planned 60 minutes).

Corticosteroids (i.e., corticosteroid therapy equivalent to 80-100 mg IV methylprednisolone) at least 30 minutes prior to subsequent infusions may be given at the Investigator's discretion.

7.9. Study Drug Accountability

Upon receipt of the study drug, the Investigator (or designee) will conduct an inventory of the supplies and verify that study drug supplies are received intact and in the correct amounts before completing a supplies receipt. The Investigator will retain a copy of this receipt at the study center and return the original receipt to the study monitor. The monitor may check the study supplies at each study center at any time during the study.

The investigational site staff must maintain a careful inventory of study drug. Study drug use will be recorded in a drug accountability record. This record will contain the following information:

- Patient number for each patient receiving study drug
- Date, quantity and dose of study drug received by the site
- Date, quantity, and dose of study drug dispensed including the calculations used to determine the dose based on patient's weight.
- Date, quantity and dose administered

At each monitoring visit, the Sponsor's clinical monitor will reconcile the information in the drug accountability record with the actual inventory of study drug at each site. All used vials of VT1021 should be disposed of according to each site's standard procedure for disposal of investigational agents. If a site is unable to directly dispose of used vials of VT1021, the monitor will arrange for disposal. At the conclusion of the study, the monitor will collect all unused vials of VT1021 for return to Vigeo Therapeutics.

7.10. Concomitant Medications and Treatments

7.10.1. Permitted Concomitant Medications and Treatments

Use of supportive care measures (i.e., growth factors, antiemetics, etc.) should follow standard (e.g., American Society of Clinical Oncology [ASCO], National Comprehensive Cancer Network [NCCN]) supportive care guidelines as listed on their websites. Patients should receive full supportive care during the study, which may include transfusion of blood or blood products, antibiotics, antiemetics, anti-diarrheals, analgesics, and other interventions as necessary. Palliative radiotherapy for pain or symptom control in the absence of disease progression is permitted. The patient may be retreated following palliative radiotherapy, providing the patient has met required criteria for starting therapy within 28 days of completing palliative radiotherapy.

> *NB:* Patients receiving denosumab or bisphosphonates initiated at least 30 days before the first dose of VT1021 may continue such treatments. Patients with carcinomas of the prostate may receive luteinizing hormone-releasing hormone (LHRH) agonists or antagonists, if initiated at least 90 days before the first dose of VT1021. Patients who do not have breast cancer may receive megestrol acetate for appetite stimulation.

Although preclinical safety testing did not identify a risk of infusion reaction with administration of VT1021, the development of mild to moderate infusion reactions (e.g., rash, pruritus) in the human study can be managed by premedication, temporary infusion interruption, reduction of the infusion rate, and symptom management (e.g., hydration, use of antihistamines, etc.). Re-challenge should be considered after complete resolution of all symptoms.

For required pre-medications, see Section 7.1.1.

Patients who receive pre-medication and subsequently experience severe infusion reactions may, at the discretion of the Clinical Investigator, be discontinued from the study.

7.10.2. Prohibited Concomitant Medications and Treatments

Patients may not receive any of the following:

- Any other investigational drug, defined as a systemically administered agent for which there is no approved indication by the United States Food and Drug Administration (US FDA). Other systemic anti-neoplastic therapies, including cytotoxic chemotherapies, hormonal therapies (with the exceptions described in Section 7.10.1), monoclonal antibodies, targeted agents, anti-angiogenic agents, immune-based therapies, or systemically administered isotopes.
- Radiation therapy for curative intent (See Section 7.10.1).
- Chronic, systemically administered glucocorticoids in doses equivalent to >5 mg prednisone daily. Topical, inhalational, ophthalmic, intraarticular, and intranasal glucocorticoids are permitted. Isolated or intermittent use of systemically administered glucocorticoids to treat complications of malignancy, use as a premedication, or as a one-time prep for an imaging procedure is permitted. If patient was on >5 mg prednisone/day equivalent, last dose must have been at least 7 days prior to the first planned dose of study drug. Exception: GBM patients may be on chronically administered glucocorticoids for the control of cerebral edema as long as the dose does not exceed 2 mg dexamethasone/15 mg prednisone/day. For GBM patients requiring larger amounts of glucocorticosteroids, consultation with, and agreement by the medical monitor is required before such patients can be enrolled.

7.11. Drug-Drug Interactions

VT1021 is a cyclic pentapeptide. It is expected to be metabolized by serum and intracellular proteases and peptidases, with the metabolic recycling of its component amino acids. In addition, the binding of VT1021 to plasma proteins is low (<20% in human plasma). As such, there are no anticipated drug-drug interactions based on data available at this time.

8. STUDY PROCEDURES

The study Schedule of Assessments and Procedures can be found in Appendix 1.

Investigators are responsible for monitoring the safety of patients who have entered this study and for alerting the clinical research organization (CRO) to any clinical event that is unexpected. The CRO will alert Vigeo Therapeutics, Inc., as per the Safety Plan.

AEs will be monitored from initial dosing of study drug up to (and including) 30 days after the last dose of study therapy.

The Investigator is responsible for appropriate medical care of study participants during the study in connection with protocol procedures.

After a study participant's completion of, or discontinuation from, the study, the Investigator remains responsible to follow, through an appropriate health care option, AEs that are serious or that caused the study participant to discontinue before completing the study.

8.1. Medical History

A medical history will be obtained during the Screening period. Only those conditions or procedures that are pertinent to the patient's participation in the study or that are ongoing (e.g., chronic concomitant medical conditions) should be listed as part of the medical history. Particular attention should be paid to the history of the patient's malignancy (e.g., date of diagnosis, initial treatment, intervening treatments).

8.2. Safety Assessments

8.2.1. Physical Examination

A complete physical exam should be conducted during the Screening period. Subsequent physical exams may be targeted and focus on areas involved with malignancy, concurrent medical conditions or with AEs (± 3 days of planned visit).

8.2.2. Vital Signs

Vital signs will include determinations of pulse, blood pressure, respiratory rate, temperature, and weight. Pulse, blood pressure, and respiratory rate should be obtained after the patient has been sitting, semi-recumbent or lying quietly for several minutes. Temperature may be obtained via the oral, axillary, aural or topical (forehead/temporal) route.

8.2.3. ECOG/Karnofsky PS

Each patient should be carefully questioned regarding performance capabilities and activities of daily living in order to determine an accurate ECOG/Karnofsky PS. Descriptions of the various ECOG/Karnofsky PS criteria can be found in Appendix 2.

8.2.4. Electrocardiograms

Twelve-lead electrocardiograms (ECGs) will be obtained in triplicate with 5-minute intervals between determinations. Each ECG should include an assessment of rate; rhythm; PR, QRS, QT and QTc intervals; and all overall assessment regarding whether the ECG is normal, abnormal

and if abnormal, whether it is clinically significant. The QTc interval should be corrected using Fridericia's formula.

8.3. Laboratory Parameters

Screening Laboratories:

During Screening, the following laboratory evaluations will be assessed:

- Serum Chemistry: Sodium, potassium, chloride, bicarbonate, blood urea nitrogen (BUN), creatinine, glucose, ALT, AST, alkaline phosphatase, total bilirubin, direct bilirubin, calcium, magnesium, phosphorous, total protein, albumin, uric acid, lipase and amylase.
- Urinalysis: dipstick determinations of pH, specific gravity, protein, glucose, ketones, blood or hemoglobin, and bilirubin. A microscopic exam should be performed if the protein or blood/hemoglobin determinations are 2+ or higher.
- Pregnancy testing may be performed via a serum beta human chorionic gonadotrophin (βHCG) level or a urine determination.
- Hematology: Complete blood count (white blood cell [WBC] count and differential, hemoglobin/hematocrit, platelet count and platelet indices [mean platelet volume [MPV]).
- Coagulation parameters: will include an aPTT/PT/INR. A prothrombin time is NOT required if the institution's laboratory only reports out an INR.

On-Study Laboratories:

During the clinical study, the following procedures should be performed as indicated:

- Hematology assessments (as noted above) will be performed within 24 hours prior to the dose of VT1021 once weekly and at follow up. Where possible, lab draws should be performed at the study clinic. Assessments may be performed at a local lab outside of the investigative site lab as long as laboratory results are reviewed by the Investigator prior to VT1021 dosing on Days 1, 8, 15, and 22. After the completion of the 2nd cycle, and at the discretion of the Clinical Investigator, Day 8 and 15 hematology testing is optional.
- If the ANC is determined to be $<1.0 \times 10^9$/L, or the platelet count is $<50 \times 10^9$/L, repeat testing should occur at intervals chosen by the Clinical Investigator in order to assure patient safety and to document duration of nadir (i.e., neutropenia, anemia, etc.). Where possible, lab draws should be performed at the study clinic. Assessments may be performed at a local lab outside of the investigative site lab as long as laboratory results are reviewed by the Investigator prior to the dose of VT1021.
- Serum chemistries (as noted above) will be performed within 24 hours prior to the dose of VT1021 once weekly and at follow up. Where possible, lab draws should be performed at the study clinic. Assessments may be performed at a local lab outside of the investigative site lab, as long as the laboratory results are reviewed by the Investigator prior to VT1021 dosing on Days 1, 8, 15 and 22. After the completion of the 2nd cycle, and at the discretion of the Clinical Investigator, Day 8 and 15 serum chemistry testing is optional.

- Urinalysis (as described above) should occur within 24 hours prior to the dose on Day 1 of each cycle and at follow up. Microscopic examination should be performed when indicated by the macroscopic analysis.
- In addition to the requirements listed above, the PI may conduct additional hematology, serum chemistry and urinalysis (and manage abnormalities identified as part of this additional testing) as medically required.

[Note: Laboratory results obtained during Screening may substitute for Cycle 1 Day 1 (C1D1) laboratory assessments if dosing is completed within 72 hours.]

8.4. Pharmacokinetic Parameters

The PK parameters of VT1021 will be determined from plasma levels obtained at various timepoints following VT1021 administration. Parameters to be calculated include maximum dose concentration (C_{max}), time to maximum dose concentration (T_{max}), area under the curve from time zero to the last measurable concentration (AUC_{0-t}), area under the curve from time zero to infinity ($AUC_{0-\infty}$), clearance, volume of distribution at steady state (V_{dss}), and the terminal elimination half-life.

The samples will be processed, stored, and shipped to the PK laboratory for analysis as detailed in the laboratory manual.

The schedule of PK blood draws is also outlined in the Schedule of Assessments and Procedures (Appendix 1).

8.4.1. Dose Escalation Cohorts

All enrolled patients will participate in the following PK evaluations during the first two cycles.

Sample times for the Dose Escalation Cohorts are presented in Table 2.

Table 2: Pharmacokinetic Sample Collection - Dose Escalation Cohorts

Pharmacokinetic Sample Collection									
	**C1D1**	**C1D4**	**C1D8**	**C1D11**	**C1D15**	**C1D18**	**C1D22**	**C1D25**	**C2D50**
Pre-dose	X	X	X	X	X	X	X	X	X
0 hr post-dose	X	X	X	X	X	X	X	X	X
2 hr post-dose	X	X	X	X	X	X	X	X	X
4 hr post-dose	X	X							X
6 hr post-dose	X	X							X
24 hr post-dose*	X	X							X

*Optional

8.4.2. Dose Expansion Cohort

Five patients enrolled in each cohort will have blood collected for PK evaluation. Sample times are presented in Table 3.

Table 3: Pharmacokinetic Sample Collection – Five Patients in Each Dose Expansion Cohort Only

	C1D1	C1D4	C2D53
Pre-dose	X	X	X
0 hr post-dose	X	X	X
2 hr post-dose	X	X	X
4 hr post-dose	X	X	X
6 hr post-dose	X	X	X

8.5. Pharmacodynamic Parameters

The PD of VT1021 will be assessed by measuring various circulating and tumor-based biomarkers, including Tsp-1 levels in PBMCs, plasma and platelets, various sub-populations of circulating immune-based cells as well as Tsp-1 expression and various other targets of VT1021 (CD36, CD47) on pre- and on-study tumor biopsies in patients whose neoplastic disease is accessible for biopsy (excluding Dose Expansion Cohort D – on-study biopsies for GBM are not required).

Patients in the Dose Expansion Cohorts will also be asked to provide results of previous biomarker testing, if applicable and available. These may include, but are not limited to, BRCA mutation status (positive or negative), MGMT methylation status (positive or negative), tumor mutation burden (TMB) score, (number of mutations per megabase), mismatch repair (MMR) deficiency results (positive or negative), microsatellite stability status (microsatellite stable [MSS] or microsatellite instable [MSI]; if MSI then low or high), and PD-L1 status (positive or negative). Results from previous biomarker testing will be evaluated for correlations to response to VT1021.

Blood Biomarkers

Tsp-1 levels will be measured by enzyme-linked immunosorbent assay (ELISA) in PBMCs, platelets, and plasma isolated from patient blood samples. The expression levels of Tsp-1 in circulation will be used to determine VT1021 activity, and the dose of VT1021 at which Tsp-1 induction plateaus may be used to identify the RP2D.

For the Dose Expansion Cohorts, immune profiling of circulating T cells by fluorescence-activated cell sorting (FACS) may also be performed to analyze the ratio of CD8+:FoxP3 positive cells among the CD3+ population, when applicable.

Myeloid-derived suppressor cells (MDSCs) isolated from predose blood from the Dose Expansion Cohorts and whole blood, PBMCs, platelets, and plasma isolated from both Escalation and Expansion Cohorts at all time points may be frozen and stored for future

exploratory PD studies. The studies may include transcriptomic, pharmacogenomic and/or proteomic analyses to identify predictive biomarkers of VT1021 response and to expand the understanding of the mechanism of action of VT1021. Participation in pharmacogenomic testing, which may include next generation or whole exome DNA sequencing, is optional.

Tumor Biopsies

In addition to PD assessments completed in blood samples, biopsies will be performed in order to understand the anti-tumor effect of VT1021. Pre- and on-study tumor biopsies (excluding Dose Expansion Cohort D- on-study biopsies for GBM are not required) will be analyzed for CD36 and CD47 expression on the surface of tumor cells. Patient biopsies will also be analyzed for the p53 target gene, Tsp-1, and for PD-L1 expression and may be analyzed to characterize the TME and tumor vascularity. Finally, Tsp-1 expression in patient biopsies will be analyzed to corroborate the results obtained from the analysis of PBMCs, platelets, and plasma and identification of the dose of VT1021 at which Tsp-1 induction plateaus. For pre- and on-study biopsies (Screening, archival, and fresh biopsies for tumor samples), a formalin fixed paraffin embedded (FFPE) block is preferred. If a tissue block is not available for the pre-VT1021 tumor sample, then slides should be submitted; see the laboratory manual for details.

All patients who are eligible for study participation must have existing tumors that are accessible for incisional, excisional or core needle biopsy, and where, in the assessment of the investigating physician, it is relatively safe and medically feasible to obtain such a biopsy. These patients may be asked to sign informed consent and provide an adequate tumor biopsy pre-VT1021 and once while receiving VT1021. In the Dose Escalation Cohorts, the on-study biopsy is collected at the end of Cycle 1 Week 4, or any time during Cycle 2. At the discretion of the Investigator, a biopsy may be taken any time after Cycle 2. In the Dose Expansion Cohorts, the on-study biopsy is collected during Cycle 2 after Cycle 2 Day 43. However, a biopsy may be obtained any time after Cycle 2 at the discretion of the Investigator. Patients enrolled in Dose Expansion Cohort D (GBM) are not required to provide on-study biopsies.

Note that "adequate tumor biopsy" is defined as ideally obtaining tumor tissue that is equivalent to ≥ 3 passes using a 16-gauge core needle (or ≥ 6 passes using an 18-gauge core needle) or providing tissue from an excisional or incisional biopsy. Fine needle aspirations (FNA), other "aspiration" samples or frozen specimens are not acceptable. FFPE blocks for both pre- and on-study biopsies from patients enrolled in the Dose Expansion Cohorts are preferred (on-study biopsies from Cohort D [GBM] patients are not required).

See the laboratory manual for details on processing of the sample tumor tissue.

Archival tumor specimens may be substituted for pre-study tumor biopsy if the specimen is adequate to perform the above testing and was obtained within 6 months of study initiation. These patients (excluding Dose Expansion Cohort D [GBM]) will still be required to undergo on-study biopsies as per schedule.

8.5.1. Dose Escalation Cohorts

All enrolled patients will participate in the following PD evaluations during the first two cycles of VT1021.

Sample times (± 10 minutes) for the Dose Escalation Cohorts are presented in Table 4.

Table 4: Pharmacodynamic Sample Collection - Dose Escalation Cohort

	Pharmacodynamic Sample Collection								
	**C1D1**	**C1D4**	**C1D8**	**C1D11**	**C1D15**	**C1D18**	**C1D22**	**C1D25**	**C2D50**
Pre-dose	X	X	X	X	X	X	X	X	X
0 hr post-dose	X	X	X	X	X	X	X	X	X
2 hr post-dose	X	X	X	X	X	X	X	X	X
4 hr post-dose	X	X							X
6 hr post-dose	X	X							X
24 hr post-dose*	X	X							X
Tumor Biopsy								X	

*Optional

8.5.2. Dose Expansion Cohorts

For the Dose Expansion Cohorts, immune profiling of circulating T cells by FACS will also be performed to analyze the ratio of CD8+:FoxP3 positive cells among the CD3+ population, when applicable.

All enrolled patients will participate in the following PD evaluations during the first two cycles of VT1021.

Sample times/assessments (± 10 minutes) for all patients enrolled in each Dose Expansion Cohort are presented in Table 5.

Table 5: Pharmacodynamic Sample Collection – All Patients in Dose Expansion Cohorts

	Blood for FACS	Blood for PD (non FACS)	Tumor Biopsy Pre- and On-study
**C1D1**			
Pre-dose	X	X	X
0 hr post-dose	-	X	-
2 hr post-dose	-	X	-
4 hr post-dose	-	X	-
6 hr post-dose	X	X	-
**C1D4**			
Pre-dose	X	X	-
0 hr post-dose	-	X	-
2 hr post-dose	-	X	-
4 hr post-dose	-	X	-
6 hr post-dose	X	X	-
**C2D53**			X[a]
Pre-dose	X	X	-
0 hr post-dose	-	X	-
2 hr post-dose	-	X	-
4 hr post-dose	-	X	-
6 hr post-dose	X	X	-

[a] Pre-study tumor biopsy may be from archival or fresh tissue and must be collected from all dose expansion patients. On-study biopsy (excluding Dose Expansion Cohort D [GBM]) is to be collected during Cycle 2 after Cycle 2, Day 43. However, biopsy may be obtained after Cycle 2 at the discretion of the Investigator.

8.6. RECIST and iRECIST or RANO Assessment

Tumors will be assessed by CT or MRI at Screening to establish extent of disease with imaging of sites to include chest, abdomen, and pelvis as well as other sites appropriate to the patient's disease status. GBM patients will require CT and / or MRI of the head and other sites as appropriate to the patient's disease status. The baseline imaging technique and sites imaged should be used for follow up scans while the patient is on study. Tumor response will be evaluated every 8 weeks ± 1 week according to the RECIST guidelines (version 1.1, Appendix

3) and iRECIST guidelines (Appendix 4), or RANO guidelines for GBM patients with iRANO modifications (Appendix 7). Other imaging procedures, as deemed appropriate by the Investigator, may be performed to assess sites of neoplastic involvement. The same method of assessment must be used throughout the study. Investigators should select target and non-target lesions in accordance with RECIST v1.1, iRECIST, or RANO (for GBM patients) guidelines. Follow-up measurements and overall response should also be in accordance with these guidelines.

Tumor assessments should be completed every 8 weeks until it is determined that the patient has progressive disease (in accordance with RECIST v1.1 and iRECIST, or RANO with iRANO modifications [for GBM patients]). The Sponsor will collect and store all tumor measurement images on all patients throughout the study; however, local radiologist and/or PI assessment will determine disease progression. A review of the scans may be performed by the Sponsor for an independent analysis of PFS and/or ORR, if necessary.

iRECIST is based on RECIST 1.1 but adapted to account for the unique tumor response seen with immunotherapeutic drugs. When clinically stable, patients should not be discontinued until progression is confirmed by the Investigator, working with local radiology, according to the rules presented in Appendix 4. This allowance to continue VT1021 despite initial radiologic progressive disease takes into account the observation that some patients can have a transient tumor flare in the first few months after the start of immunotherapy, and then experience subsequent disease response.

A description of the adaptations and iRECIST process is provided in Appendix 4, with additional detail in the iRECIST publication (Seymour et al, 2017). iRECIST will be used by the Investigator to assess tumor response and progression and to make treatment decisions.

8.6.1. Tumor Biomarker Assessment

If applicable, a blood sample for measurement of tumor biomarkers will be obtained during Screening, then every 8 weeks ±1 week in association with tumor scans, and at the end of study (EOS) visit as specified in the Schedule of Assessments and Procedures (Appendix 1). Blood for tumor biomarker assessment is to be collected within ±7 days of tumor measurements. Blood will be collected every 8 weeks for analysis of appropriate tumor biomarkers. Where possible, lab draws should be performed at the study clinic. Assessments may be performed at a local lab outside of the investigative site lab as long as laboratory results are reviewed by the Investigator. Tumor markers to be measured for specific indications in Dose Expansion Cohorts include:

- Ovarian: CA125
- Pancreatic: CA-19-9
- TNBC: CA 15-3
- Basket high-CD36: appropriate biomarker per indication

8.7. Preliminary Efficacy Parameters

8.7.1. Best Overall Response

Best overall response will be based on the RECIST 1.1 criteria (Appendix 3), iRECIST (Appendix 4), or RANO criteria with iRANO modifications (for GBM patients; Appendix 7) and will represent the best response compared to the extent of disease assessment at Screening. The Investigator will use all appropriate radiologic procedures to document areas of involvement. The use of physical findings to document areas of disease should primarily be limited to involvement of the skin and other superficial structures. Each area found to contain neoplastic disease during Screening must be evaluated at each follow-up assessment.

8.7.2. Duration of Response

The duration of response is the interval between the first documented response (complete response [CR] or partial response [PR], whichever status is recorded first) until the first date that recurrence or progressive disease is objectively documented, taking as reference for progressive disease the smallest measurements recorded since VT1021 started.

8.7.3. studProgression-Free Survival

Progression-free survival is the interval between the first day of VT1021 and documented progression of disease (RECIST or RANO progression or clinical progression) or death from any cause. Patients who may be lost to follow up will be censored on the last day that they were known to not have progressed.

9. SAFETY REPORTING

9.1. Definitions

9.1.1. Adverse Event

An AE is any untoward medical occurrence associated with the use of a drug in humans, whether or not considered drug related. An AE can be any unfavorable and unintended sign (e.g., an abnormal laboratory finding), symptom, or disease temporally associated with the use of a drug and does not imply any judgment about causality.

9.1.2. Suspected Adverse Reaction

A suspected adverse reaction is any AE for which there is a reasonable possibility that the drug caused the AE. For the purpose of Investigational New Drug (IND) safety reporting, "reasonable possibility" means there is evidence to suggest a causal relationship between the drug and the AE. Inherent in this definition is the need for the Sponsor to evaluate the available evidence and make a judgment about the likelihood that the drug actually caused the AE. An adverse reaction is any AE caused by a drug. Adverse reactions are a subset of all suspected adverse reactions where there is reason to conclude that the drug caused the event.

9.1.3. Unexpected Adverse Event

An AE or adverse reaction is considered "unexpected" if it is not listed in the Investigator brochure or is not listed at the specificity or severity that has been observed. As an example, under this definition, hepatic necrosis would be unexpected (by virtue of a greater severity) if the Investigator brochure referred only to elevated hepatic enzymes or hepatitis. Similarly, cerebral thromboembolism and cerebral vasculitis would be unexpected (by virtue of greater specificity) if the Investigator brochure listed only cerebral vascular accidents. When new AE information is received, it is the Sponsor's responsibility to determine whether the event is unexpected.

9.1.4. Serious Adverse Event

An AE is considered serious if, in the view of either the Investigator or Sponsor, it results in any of the following outcomes:

- Death
- A life-threatening AE
- Inpatient hospitalization or prolongation of existing hospitalization
- A persistent or significant incapacity or substantial disruption of the ability to conduct normal life functions
- A congenital anomaly/birth defect

Important medical events that may not result in the above may be considered serious when, based upon appropriate medical judgment, they may jeopardize the patient and may require medical or surgical intervention to prevent one of the outcomes listed in this definition.

An AE is considered life threatening if, in the view of either the Investigator or Sponsor, its occurrence places the patient at immediate risk of death. It does not include an AE or suspected adverse reaction that, had it occurred in a more severe form, might have caused death.

Hospitalizations for elective or planned purposes, for the administration of protocol therapy, for the administration of blood products or ancillary therapies, for procedures such as endoscopy or placement of intravascular access device, for purposes of disposition or respite care or that are less than 24 hours in duration would not fulfill the above definition of serious. Complications arising from elective or planned hospitalizations that result in prolonged hospitalization would be considered SAEs.

9.1.5. Dose Limiting Toxicity

A DLT is defined as any of the following AEs that is considered related to VT1021 during Cycle 1.

Non-hematologic Toxicity:

- Grade 3 nausea, vomiting, and/or diarrhea that lasts > 48 hours despite maximum medical support.
- Grade 3 electrolyte imbalance that does not correct within 48 hours to < Grade 2 despite maximal medical intervention.
- Grade 3 fatigue that does not improve to ≤ Grade 2 within 5 days.
- Grade 3 QTc interval prolongation with a ≥ 60 ms change from baseline.
- Any other Grade ≥ 3 AE considered at least “possibly related” to study drug(s), **except**:
    - Non-hematologic laboratory Grade 3 AE that is asymptomatic and/or rapidly reversible (returned to baseline or to Grade ≤ 1 within 7 days) unless identified as clinically relevant by the Investigator.
- Any death not clearly due to underlying disease or an extraneous cause.
- Fulfilment of Hy’s law criteria: Elevated ALT or AST by 3-fold or greater above the upper limit of normal with concomitant elevation of serum total bilirubin of greater than 2× ULN, without findings of cholestasis (defined as serum alkaline phosphatase activity less than 2× the ULN) and no other reason can be found to explain the combination of increased aminotransferase and serum total bilirubin, such as viral hepatitis, alcohol abuse, ischemia, preexisting liver disease, or another drug capable of causing the observed injury.
- ≥ Grade 3 amylase or lipase that is not associated with symptoms or associated manifestations of pancreatitis.

Hematologic Toxicity:

- Grade 4 neutropenia lasting > 7 days in the absence of growth factor support.
- Grade 3 neutropenia of any duration associated with fever ≥ 38.5°C.
- Grade 3 thrombocytopenia with bleeding.
- Any other Grade 4 hematologic toxicity.

Other Toxicity:

- Three or more missed doses within Cycle 1 due to a possible study drug(s)-related toxicity.
- Inability to administer scheduled cycle of VT1021 within 14 days of the scheduled start of Cycle 2 due to a possible study drug-related toxicity.

If AEs that fulfill DLT criteria occur after Cycle 1, a meeting will occur to discuss the applicability of such events on continued enrollment/dosing in the current cohort as well as in future cohorts.

9.2. Grading of Adverse Events

All AEs will be graded using CTCAE Version 5.0 (https://ctep.cancer.gov/protocoldevelopment/electronic_applications/docs/CTCAE_v5_Quick_Reference_5x7.pdf)

For adverse events that are not listed in the CTCAE, the following severity grading classification should be employed:

- **Grade 1:** A mild AE; transient or mild discomfort; no limitation in activity; no medical intervention or therapy required
- **Grade 2:** A moderate AE; mild to moderate limitation in activity; some assistance may be needed; no or minimal medical intervention or therapy required
- **Grade 3:** A severe AE; marked limitation in activity; some assistance usually required; medical intervention or therapy required; hospitalization may be required
- **Grade 4:** A life-threatening AE; the patient was in immediate danger of dying from the event as it occurred; extreme limitation in activity; significant assistance required; significant medical intervention or therapy required
- **Grade 5:** A fatal event

9.3. Determination of Causality

The Investigator must attempt to determine if an AE is in some way related to the use of the study drug. This relationship should be described as follows:

- **Unrelated:** The event has no temporal relationship to study drug administration; or there is a reasonable causal relationship between the AE and another drug, concurrent disease or circumstance.
- **Unlikely:** The event has a temporal relationship to drug administration which makes a causal relationship improbable, and in which other drugs, chemicals or underlying disease provide plausible explanations.
- **Possible:** The event follows a reasonable temporal sequence from administration of the study drug and the event follows a known response pattern to the study drug *BUT* the event could have been produced by an intercurrent medical condition which, based on the pathophysiology of the condition, and the pharmacology of the study drug, would be unlikely related to the use of the study drug *or* the event could be the effect of a concomitant medication.

- **Probable:** The event follows a reasonable temporal sequence from administration of the study drug and the event follows a known response pattern to the study drug *AND* the event cannot have been reasonably explained by an intercurrent medical condition *or* the event cannot be the effect of a concomitant medication.
- **Definite:** The event follows a reasonable temporal sequence from administration of the study drug, the event follows a known response pattern to the study drug and based on the known pharmacology of the study drug, the event is clearly related to the effect of the study drug.

For the purposes of expedited reporting (see Section 9.4.1), causality assessments of possibly, probably, or definitely related will be considered "related."

9.4. Adverse Event Reporting

AE reporting will begin on the date the patient provides informed consent to participate in the study and will be documented in the database. In particular, SAEs associated temporally with study procedures prior to first exposure to study medication (including biopsies) must be collected and reported immediately. The Investigator should elicit information regarding the occurrence of AEs through open-ended questioning of the patient, physical examination and review of laboratory results.

All AEs, whether serious or not, will be recorded in the source documents and the AE page of the electronic case report form (eCRF) (except as noted below). All new events, as well as those that worsen in intensity or frequency relative to baseline, which occur after first administration of study drug through 30 days following the last dose of study drug, must be recorded. AEs should be followed through resolution, where possible. Events occurring from the time of signing informed consent until the beginning of protocol therapy will be considered a part of the patient's medical history. SAEs that are felt by the Investigator to be related to protocol therapy may be reported beyond the 30-day window following last dose of protocol therapy. AEs will be recorded at each patient interaction with study site staff.

Wherever possible, a specific disease or syndrome rather than individual associated signs and symptoms should be identified by the Investigator and recorded on the CRF. However, if an observed or reported sign or symptom is not considered a component of a specific disease or syndrome by the Investigator, it should be recorded as a separate AE on the CRF.

Appropriate CTCAE terms for AEs should be used whenever possible. If an appropriate CTCAE term is not available, then the verbatim term should be documented.

Laboratory, vital signs, ECG abnormalities, or progressive disease are to be recorded as AEs only if they are medically/clinically relevant: symptomatic, requiring corrective treatment, leading to discontinuation and/or fulfilling a seriousness criterion.

Questions sometimes arise about when an abnormal laboratory value should be reported as an AE. Although this decision is at the discretion of the Investigator, an abnormal laboratory value should be reported as an AE if it is clinically significant; if it is associated with clinical sequelae; if it is an SAE or important medical event; if it requires a discontinuation or change in protocol therapy; if it requires medical intervention; or if it results in study discontinuation.

If a laboratory abnormality is one component of a diagnosis or syndrome, then only the diagnosis or syndrome should be recorded as an AE. It is more advantageous to report a laboratory

abnormality as a clinical event with a medical term rather than an abnormal laboratory result per se (record thrombocytopenia rather than decreased platelet count).

Information to be reported in the description of each AE includes:

- A medical diagnosis of the event (if a medical diagnosis cannot be determined, a description of each sign or symptom characterizing the event should be recorded)
- The date of onset of the event
- The date of resolution of the event
- Grade of the event
- A determination of whether the event is serious or not
- Action taken: none; change in the study drug administration (e.g., temporary interruption in dosing); drug treatment required; non-drug treatment required; hospitalization or prolongation of hospitalization required (complete SAE page); diagnostic procedure performed; patient discontinued from the study (complete Final Visit Section of the case report form)
- Outcome: resolved without sequelae; resolved with sequelae; event resolving; event ongoing; patient died (notify the Sponsor immediately and complete the SAE page and the Final Visit section of the case report form).

9.4.1. Expedited Reporting of Adverse Events

All unexpected fatal or life-threatening suspected adverse reactions, including DLTs, must be reported to the Sponsor or Sponsor’s representative and the Sponsor’s Medical Monitor immediately by telephone or e-mail followed by submitting a completed SAE form within 24 hours of the investigative site's awareness. If full information is not known additional follow up by the Investigator will be required.

All other SAEs must be reported to the Sponsor or Sponsor’s representative within 24 hours by phone, email or fax followed by submitting a completed SAE form via e-mail or fax within 48 hours whether full information regarding the event is known or not. If full information is not known additional follow up by the Investigator will be required. Faxed reports will be sent to:

Pharmacovigilance:

Medical Monitors

Kelly Curtis, MD
Emergency Contact Telephone: 602-245-3004
Email: Kelly.Curtis@syneoshealth.com

Lou Vaickus MD
Emergency Contact Telephone: 857-919-2493
Email: lou.vaickus@vigeotherapeutics.com

SAE Reporting Information

Fax: US toll free – 1-877-464-7787

SAE reporting email in case of fax failure:

safetyreporting@syneoshealth.com

All SAEs will be evaluated by the Sponsor's Medical Monitor. If meeting the requirements for expedited reporting, the Sponsor will report the AE to the appropriate health authorities and to all Investigators. The Investigator must report all SAEs reported to regulatory authorities in an expedited manner to the Institutional Review Board (IRB).

Follow-up SAE reports are required when there is a significant development in the case. Examples of significant developments include: updates from diagnostic imaging or laboratory testing, new information from specialist consultation, or a change in the clinical status of the patient (either improved or worsened). Follow-up reports for significant developments are required until the case is considered to be concluded (as indicated by resolution of the event such that it is no longer "serious" or as indicated by other means (i.e., patient discharge, patient death).

9.5. Adverse Event Follow Up

AEs must be followed until resolution, stabilization, or return to a baseline state. For SAEs, there is often insufficient information to completely describe the event at time of reporting. Information regarding such an event should continue to be collected until the nature of the event, its treatment and its outcome are fully described. The Sponsor or the Sponsor's representative will follow up with the investigational sites to collect additional information regarding an AE as necessary.

9.6. Pregnancy

Patients who are sexually active and able to bear or beget children must take the appropriate precautions not to (for females) become pregnant, or (for males) to prevent pregnancy while enrolled in this study. These patients must agree to use two reliable forms of contraception during the study, for example, oral contraceptive and condom, intrauterine device and condom, diaphragm with spermicide and condom. This should be continued for at least 90 days after the last dose of VT1021.

All patients of childbearing potential, who are sexually active, must have a negative serum pregnancy test within 1 week prior to C1D1 and at each subsequent cycle. Patients found to be pregnant must not be enrolled (or continue VT1021) in the study and must not receive VT1021. In addition, all women of childbearing potential should be instructed to contact the Investigator immediately if they suspect pregnancy has occurred at any time during study participation. The Investigator must immediately notify the CRO of this occurrence.

10. STATISTICAL METHODS

10.1. Sample Size Determination

No sample size justification was performed for the Dose Escalation Phase in this study. The Dose Escalation Phase of the study will be completed upon enrollment of approximately 20-30 patients. Each expansion cohort will enroll no fewer than 6 patients to establish a "safety cohort" for that indication.

10.2. Study Populations

Analysis populations will be determined based on the evaluable nature of the patients. All patients signing informed consent will be considered enrolled. Patients who are enrolled but not dosed will be listed only.

- Safety Population (SAF): Those patients who receive at least one dose of VT1021 will be included in the safety analysis.
- Per Protocol Population (PP): All patients who receive at least one dose of VT1021 and have at least one post-baseline tumor assessment scan. The PP patients will be used for the primary analysis for each cohort.
- Evaluable: All patients completing Cycle 1 and receiving at least 75% of the planned doses for the cycle, where missed doses must not have been omitted due to VT1021-related toxicity.
- Pharmacokinetic Population (PKP): SAF patients who have evaluable PK samples.
- Pharmacodynamic Population (PDP): SAF patients that have evaluable PD samples.

10.3. Statistical Analysis

10.3.1. General Considerations

Data will be cleaned according to the appropriate data management plan. Sites will update the electronic case report form as quickly and accurately as possible. Data reviews will occur on a regular basis. Upon completion of the final visit for the last patient all patient data will be cleaned and locked. A statistical analysis plan (SAP) containing details of statistical methods will be finalized prior to database lock.

Descriptive statistics will be performed as appropriate. Continuous parameters will include at a minimum: n, mean, median, and standard deviation, whereas categorical parameters will be summarized by frequency and percentages. Two-sided 95% confidence interval may be calculated when appropriate. No inferential analysis will be performed in this study. If a p-value is provided it will be used for descriptive purposes only and not to draw inferences on the data.

Table displays will be performed by dose level at a minimum. If enough data exists data may be displayed by dose level within an indication. All analysis will be performed using SAS version 9.4 or higher.

10.3.2. Sample Size Determination

No sample size calculations were performed for the dose escalation portion of this study. The Dose Expansion has multiple cohorts to determine the proof of concept. Each cohort will be analyzed separately, however the sample size calculation will be the same across each. All cohorts will be analyzed using the per protocol set.

H_0: ORR ≤ 0.15 versus H_A: ORR > 0.375 will be tested using a 1-sided significance level at 0.10. A total of 15 patients is required to provide 80% power to reject the null hypothesis, if not true. Assuming an attrition rate of 15% an enrollment of approximately 16-17 patients may be required to obtain 15 evaluable patients. Therefore, the number enrolled in total across all five cohorts will be approximately 80-85.

10.3.3. Patient Disposition, Demographics and Baseline Characteristics

Number of patients enrolled, dosed, and who discontinue VT1021 and the study will be summarized by reason. All study populations will be summarized.

Demographic parameters such as age, race, ethnicity, weight, and height will be summarized. Cancer diagnosis, prior regimen, medical history will be summarized.

10.3.4. Exposure to VT1021

The number of patients receiving VT1021 will be summarized overall and by cycle. The amount of VT1021 will be summarized by total and mean dose.

Dose reductions, holds, interruption, and missing doses will be counted and summarized overall and by cycle.

10.3.5. Preliminary Efficacy Analyses

The primary efficacy analysis on each Dose Expansion Cohort will be performed using an exact Clopper-Pearson test on ORR (PR + CR) and 95% exact confidence intervals will be analyzed based on RECIST v1.1 or other relevant criteria (e.g., RANO for GBM patients or CA 125 level in ovarian cancer patients as described in the Gynecologic Cancer InterGroup [GCIG]).

DCR is defined DCR = PR + CR + stable disease (SD), and will be described using RECIST v1.1. In this study, SD will be counted in DCR if its duration is ≥ 4 months or 2 tumor scans, whichever is longer. The estimated rate and exact confidence intervals will be displayed. The estimated rate will be analyzed using an exact Clopper-Pearson test. PFS will also be analyzed. PFS is defined as the time from first dose to the first documented progression of disease using RECIST v1.1 (based on Investigator assessment) or death from any cause, whichever comes first. PFS for patients with specific indications will be based upon the criteria relevant to that specific indication. PFS will be displayed using Kaplan-Meier estimates.

10.3.6. Safety Analyses

Treatment-emergent adverse events (TEAEs) will be summarized by Medical Dictionary for Regulatory Activities (MedDRA) system organ class and preferred term. Additional summaries of SAEs, study drug-related AEs, serious TEAEs, and where appropriate, Grade 3 and higher TEAEs. TEAEs will be displayed by grade. Separate listings will be presented for different categories such as SAEs/Deaths, DLTs, etc.

Laboratory analytes will be analyzed using shifts from baseline using the CTCAE version 5.0. When appropriate graphical displays will be displayed. Vital signs will be similarly analyzed.

10.3.7. Interim Analyses

No formal interim analyses are required for this study.

10.3.8. Pharmacokinetic, Pharmacodynamic and Biomarker Analyses

Serum/plasma samples collected from patients will be analyzed for the concentrations of VT1021 to estimate PK parameters. Standard non-compartmental analysis method will be used to determine the PK parameters of VT1021 including C_{max}, T_{max}, AUC_{0-t}, $AUC_{0-\infty}$, clearance, V_{dss}, and the terminal elimination half-life. The analyzed PK parameters will be summarized using descriptive statistics, including the median, geometric mean and 95% confidence intervals around parameter estimates by dose level.

The PD of VT1021 will be assessed by measuring various circulating and tumor-based biomarkers, including Tsp-1 levels in PBMCs, plasma, and various sub-populations of circulating immune-based cells. Tsp-1 and other targets of VT1021 (CD36, CD47) will be also be assessed on pre- and on-study tumor biopsies in patients whose neoplastic disease is accessible for biopsy (excluding Dose Expansion Cohort D-GBM, as on-study biopsy collection for this cohort is not required).

Tsp-1 levels will be summarized by observed and change from baseline. Change from baseline will be analyzed using a signed rank test. The ratio of CD8+:FoxP3 positive cells among the CD3+ population will be summarized by observed and change from baseline, when applicable. The expression levels of Tsp-1 in circulating PBMCs and plasma will be used to determine VT1021 activity, and the dose of VT1021 at which Tsp-1 induction plateaus may be used to identify the RP2D.

All PD parameters will be summarized. When applicable, correlation and testing will be performed to evaluate the relationship to outcomes (e.g., ORR). Inference testing will be used in a more descriptive manner in order to plan future studies and understanding of the compound.

11. ETHICAL ASPECTS

11.1. Ethical Conduct of the Study and IRB Oversight

The study will be conducted according to the principles of the most recent version of the Declaration of Helsinki, the International Conference on Harmonization (ICH) Guidance on Good Clinical Practice (GCP) and the requirements of all local regulatory authorities regarding the conduct of clinical studies and the protection of human patients.

The Investigator will submit the protocol, the Investigator's Brochure, informed consent, and any other material used to inform patients about the study to the IRB for approval prior to enrolling any patient into the study. The IRB should be duly constituted according to applicable regulatory requirements. Approval must be in the form of a letter signed by the Chairperson of the IRB or the Chairperson's designee, must be on IRB stationery and must include the protocol by name and/or designated number. If an Investigator is a member of the IRB, the approval letter must stipulate that the Investigator did not participate in the final vote, although the Investigator may participate in the discussion of the study. The Investigator will also inform the IRB of any SAEs that the Sponsor reports to regulatory authorities and will provide to the IRB a final summary of the results of the study at the conclusion of the study.

11.2. Informed Consent

No study related procedures will be performed until a patient or a patient's legal representative has given written informed consent. The Sponsor will provide to the Investigator a sample informed consent document that includes all the requirements for informed consent according the ICH GCP and US FDA guidelines (21 Code of Federal Regulations [CFR] 50). However, it is up to the Investigator to provide a final informed consent that may include additional elements required by the Investigator's institution or local regulatory authorities. The informed consent document must clearly describe the potential risks and benefits of the study, and each prospective participant must be given adequate time to discuss the study with the Investigator or site staff and to decide whether to participate. Each patient who agrees to participate in the study and who signs the informed consent will be given a copy of the signed, dated and witnessed document. A second copy of the signed, dated and witnessed informed consent document will be retained by the Investigator in the study files.

11.3. Patient Confidentiality

It is the responsibility of the Investigator to ensure the confidentiality of all patients participating in the study and all their protected health information is maintained. Any source or other documents submitted to the Sponsor must never contain the name of a study participant. Each patient in the study will be identified by a unique identifier that will be used on all eCRFs and any other material submitted to the Sponsor. Any identifying information must be kept in a secure location with access limited to the study staff directly participating in the study. All protected health information collected and processed for the purposes of this study should be managed by the Investigators and their staffs with adequate precautions to ensure the confidentiality of those data, and in accordance with the Health Insurance Portability and Accountability Act (HIPAA) and any other applicable national and/or local laws and regulations on personal data protection.

Personal medical information may be reviewed by representatives of the Sponsor, of the IRB or of regulatory authorities in the course of monitoring the progress of the study. Every reasonable effort will be made to maintain such information as confidential.

The results of the study may be presented in reports, published in scientific journals or presented at medical meetings; however, patient names will never be used in any reports about the study.

12. STUDY ADMINISTRATION

12.1. Monitoring

A Clinical Research Associate (CRA) will make regularly scheduled trips to the investigational sites to review the progress of the study, in addition to performing remote monitoring in accordance with site guidelines. The actual frequency of monitoring trips will depend on the enrollment rate and performance at each site. At each visit, the CRA will review various aspects of the study including, but not limited to, Screening and enrollment logs, compliance with the protocol and with the principles of GCP, completion of case report forms, source data verification, study drug accountability, and storage, facilities and staff.

During scheduled monitoring visits, the Investigator and the investigational site staff must be available to meet with the study monitor to discuss the progress of the study, make necessary corrections to case report form entries, respond to data clarification requests and respond to any other study-related inquiries of the monitor.

In addition to the above, representatives of the Sponsor’s auditing staff or government inspectors may review the conduct/results of the study at the investigational site. The Investigator must promptly notify the Sponsor of any audit requests by regulatory authorities.

12.2. Data Collection and Data Quality Assurance

Data for this study will be captured via a web-based electronic data capture (EDC) system. An audit trail will maintain a record of initial entries and changes made; reasons for change; time and date of entry; and user name of person who made the change.

For each patient entered, an eCRF must be completed. The PI or authorized delegate from the study staff will electronically sign the eCRFs. If a patient interrupts study drug or is permanently withdrawn from the study drug, the reason for interruption or discontinuation must be noted in the eCRF. The Investigator should ensure the accuracy, completeness, and timeliness of the data reported to the Sponsor in the eCRFs and in all required reports.

Case report forms will be checked for correctness against source document data by the Sponsor’s monitor. If any entries into the eCRF are incorrect or incomplete, the monitor will ask the Investigator or the study site staff to make appropriate corrections. Any discrepancies noted in the data will be queried via a data clarification process within the EDC system.

12.3. Pre-Study Documents

Prior to initiating the study, the Investigator will provide to the Sponsor the following documents:

- A signed FDA Form 1572
- A current (within 2 years), dated and signed curriculum vitae for the PI and each sub-Investigator listed on the FDA Form 1572
- A copy of the Investigator’s medical license from the state in which the study is being conducted; the Investigator’s medical license number on the Investigator’s c.v. will suffice as evidence of a license to practice medicine

- A letter from the IRB stipulating approval of the protocol, the informed consent document and any other material provided to potential study participants with information about the study (e.g., advertisements)
- A copy of the IRB approved informed consent document
- Current IRB membership list for IRB's without a multiple project assurance number or an IRB organization number under the Federal Wide Assurance program (www.ohrp.osophs.dhhs.gov)
- A signed Investigator Protocol Agreement
- A completed financial disclosure form
- Current laboratory certification for the reference laboratory
- A list of current laboratory normal values for the reference laboratory
- Curriculum vitae of the laboratory director.

12.4. Protocol Amendments

The protocol will only be amended by the Investigator with the agreement of the Sponsor and the IRB. Changes to the protocol must be in the form of a written amendment; changes other than those of a simple administrative nature (e.g., a new telephone number for a Medical Monitor) must be submitted by the Investigator to the local IRB and such amendments will only be implemented after written approval of the requisite IRB. All amendments will also be submitted to local regulatory authorities by the Sponsor as required by local regulation.

Protocol changes to eliminate an immediate hazard to a study patient may be implemented by the Investigator immediately. The Investigator must then immediately inform the IRB and the Sponsor who will immediately notify local regulatory authorities.

If an amendment substantially alters the study design or increases the potential risk to the patient, the consent form must be revised and submitted to the IRB for review and approval; the revised form must be used to re-consent patients currently enrolled in the study if they are affected by the amendment; and the new form must be used to obtain consent from new patients prior to enrollment.

12.5. Records Retention

The Investigator will retain the records of the study for 15 years, or for 2 years following the date that a marketing application for the study drug is approved, or if no marketing application is filed, or if such an application is not approved, for 2 years after the IND has been closed. The Sponsor will notify Investigators when retention of study records is no longer required. All study records must be maintained in a safe and secure location that allows for timely retrieval, if needed.

Study records that must be retained include copies of case report forms, signed informed consents, correspondence with the IRB, study drug dispensing and inventory records, source documents, clinic charts, medical records, laboratory results, radiographic reports and Screening/enrollment logs.

Should the Investigator relocate, retire, or should there be any changes in the archival arrangements for the study records, the Sponsor must be notified. The responsibility for maintaining the study records may be transferred to another suitable individual, but the Sponsor must be notified of the identity of the individual assuming responsibility for maintaining the study records and the location of their storage. If no other individual at the investigational site is willing to assume this responsibility, the Sponsor will assume responsibility for maintaining the study records.

12.6. Reporting and Publications

At the conclusion of the study, a clinical study report will be prepared by the Sponsor or its designee. All information and data obtained in the course of the study are the property of the Sponsor and are considered confidential. Publication of information and data is governed by the contract between the Sponsor and the investigative instruction. Authorship of any publications will be based on the criteria of the International Committee of Medical Journal Editors (www.icmje.org). The ICMJE recommends that authorship be based on the following 4 criteria:

- Substantial contributions to the conception or design of the work; or the acquisition, analysis, or interpretation of data for the work; AND
- Drafting the work or revising it critically for important intellectual content; AND
- Final approval of the version to be published; AND
- Agreement to be accountable for all aspects of the work in ensuring that questions related to the accuracy or integrity of any part of the work are appropriately investigated and resolved.

12.7. Study Discontinuation

The Sponsor may discontinue the study at any time.

The study may be terminated based on the following criteria:

- Toxicity at the lowest dose level is determined to be higher than the acceptable toxicity level.
- Targeted sample size has been reached (total of 20 to 30 patients in escalation cohorts, and total of approximately 80-85 in expansion cohorts).

Other reasons for terminating the study early may be applicable depending upon the toxicity and/or safety considerations.

Reasons for early study discontinuation include, but are not limited to:

- Investigator noncompliance with the protocol, good clinical practice, and/or regulatory requirements.
- Problems in manufacturing study drug.
- Inadequate enrollment.
- Unexpected safety concerns.
- Sponsor’s decision to modify or discontinue the development of VT1021.

- Request to discontinue the study by a regulatory authority.

The Sponsor will promptly inform all Investigators and the FDA and/or local regulatory authorities, if the study is suspended or terminated for any reason. The Investigator will promptly notify the IRB/Ethics Committee if the study is suspended or terminated.

13. REFERENCES

Baker LH, Rowinsky EK, Mendelson D, et al. Randomized, phase II study of the thrombospondin-1-mimetic angiogenesis inhibit ABT-510 in patients with advanced soft tissue sarcoma. J Clin Oncol 26:5583-5588, 2008

Bhattacharjee A, Richards WG, Staunton J, et al. Classification of human lung carcinomas by mRNA expression profiling reveals distinct adenocarcinoma subclasses, Proc Nat Acad Sci USA, 98: 13790-5, 2001.

Carvelli L, Libin Y, Morales CR. Prosaposin: a protein with differential sorting and multiple functions. Histol Histopathol 3);647-660, 2015

Catena R, Bhattacharya N, El Rayes T, et al. Bone marrow-derived $Gr1^+$ cells can generate a metastasis-resistant microenvironment via induced secretion of thrombospondin-1. Cancer Discovery 3:578-589, 2013

Chen X, Cheung ST, So S, et al. Gene expression patterns in human liver cancers, Molecular Biology of the Cell, 13: 1929-39, 2002.

ClinicalTrials.gov (a). A trial of TTI-621 for Patients with Hematologic Malignancies. https://www.clinicaltrials.gov/ct2/show/NCT02663518?term=CD47&rank=4

ClinialTrials.gov (b). CAMELLIA: Anti-CD47 Antibody Therapy in Relapsed/Refractory Acute Myeloid Leukaemia. https://www.clinicaltrials.gov/ct2/show/NCT02678338?term=CD47&rank=1

Dawson DW, Pearce SF, Zhong R, Silverstein RL, Frazier WA, Bouck NP. CD36 mediates the In vitro inhibitory effects of thrombospondin-1 on endothelial cells. J Cell Bio, 138: 707-17, 1997.

Ebbinghaus S, Hussain M, Tannir N, et al. Phase 2 study of ABT-510 in patients with previously untreated advanced renal cell carcinoma. Clin Cancer Res 13:6689-6695, 2007

Ellingson BM, Bendszus M, Boxerman J, et al. Consensus recommendation for a standardized Brain Tumor Imaging Protocol in clinical trials. Neuro Oncol. 9: 1188-1898, 2015

Hong Y, Downey T, Eu KW, Koh PK, Cheah PY. A 'metastasis-prone' signature for early-stage mismatch-repair proficient sporadic colorectal cancer patients and its implications for possible therapeutics. Clinical & Experimental Metastasis, 27: 83-90, 2010.

Kang S-Y, Halvorsen OJ, Gravdal K, et al. Prosaposin inhibits tumor metastasis via paracrine and endocrine stimulation of stromal p53 and Tsp-1. PNAS 106:12115-12120, 2009.

Kaur S, Martin-Manso G, Pendrak ML, et al. Thrombospondin-1 inhibits VEGF receptor-2 signaling by disrupting its association with CD47. J Biol Chem 285:38923-38932, 2010.

Molckovsky A and Siu LL. First-in-class, first-in-human phase I results of targeted agents: Highlights of the 2008 American Society of Clinical Oncology meeting. J Hematol Oncol 1:20-28, 2008.

Russell S, Duquette M Liu J, et al. Combined therapy with thrombospondin-1 type repeats (3TSR) and chemotherapy induces regression and significantly improves survival in a preclinical model of advanced stage epithelial ovarian cancer. FASEB J 29:576-588, 2015.

Seymour L, Bogaerts J, Perrone A, et al. iRECIST: guidelines for response criteria for use in trials testing immunotherapeutics. Lancet Oncol 2017;18(3): e143–e152.

Wang S, Blois A, el Rayes T, et al. Development of a prosaposin-dervied therapeutic cyclic peptide that targets ovarian cancer via the tumor miroenvironment. Sci Transl Med 8:329ra34, 2016

Wen PY, Macdonald DR, Reardon DA, et al. Updated response assessment criteria for high-grade gliomas: response assessment in neuro-oncology working group. J Clin Oncol. 2010; 28(11):1963-1972.

Yoshihara, K, Tajima A, Komata D, et al. Gene expression profiling of advanced-stage serous ovarian cancers distinguishes novel subclasses and implicates ZEB2 in tumor progression and prognosis. Cancer Sci, 100: 1421-8, 2009.

Zhang X, Kazerounian S, Duquette M, et al. Thrombospondin-1 modulates vascular endothelial growth factor activity at the receptor level. FASEB J 23:3368-3376, 2009.

14. APPENDICES

Appendix 1: Schedule of Assessments and Procedures....79
Appendix 2: The ECOG and Karnofsky Performance Scores....83
Appendix 3: RECIST 1.1 Criteria....85
Appendix 4: Description of the iRECIST Process for Assessment of Disease Progression....89
Appendix 5: CTCAE v5.0 Criteria....92
Appendix 6: The Declaration of Helsinki....93
Appendix 7: The RANO Criteria for Assessment of Glioblastoma Patients....94

Appendix 1: Schedule of Assessments and Procedures

Procedure	Screen (D-28 to D-1)	Treatment Period																								EOS	30-Day FU[17]
		Cycle 1								Cycle 2								Cycle 3+									
		Wk 1		Wk 2		Wk 3		Wk 4		Wk 5		Wk 6		Wk 7		Wk 8		Wk 9		Wk 10		Wk 11		Wk 12			
		D 1	D 4	D 8	D 11	D 15	D 18	D 22	D 25	D 29	D 32	D 36	D 39	D 43	D 46	D 50	D 53	D 57	D 60	D 64	D 67	D 71	D 74	D 78	D 81		
Informed Consent[1]	X																										
Inclusion/Exclusion Criteria	X																										
Medical History[2]	X																										
Physical Exam	X	X[12]								X[12]								X[12]							X[12]	X[12]	X[12]
Vital Signs[3]	X	X		X		X		X		X		X		X		X		X		X		X		X	X	X	X
Karnofsky/ECOG Performance Score	X	X								X								X							X	X	X
Electrocardiogram[4]	X	X[4]								X[4]																	
Complete Blood Count[5]	X	X		X		X		X		X		X		X		X		X							X	X	X
Serum Chemistries[6]	X	X		X		X		X		X		X		X		X		X							X	X	X
Coagulation Parameters[7]	X	X																									
Urinalysis[8]	X									X								X								X	X
Pregnancy Test[9]	X									X								X									
Extent of Disease Determination[10]	X																X[10]									X	
Adverse Event Reporting	X	X	X	X	X	X	X	X	X	X	X	X	X	X	X	X	X	X	X	X	X	X	X	X	X	X	X
Prior/Concomitant Medications	X	X	X	X	X	X	X	X	X	X	X	X	X	X	X	X	X	X	X	X	X	X	X	X	X	X	X
Administration of VT1021		X	X	X	X	X	X	X	X	X	X	X	X	X	X	X	X	X	X	X	X	X	X	X	X		

Procedure	Screen (D-28 to D-1)	Treatment Period																								EOS	30-Day FU[17]
		Cycle 1								Cycle 2								Cycle 3+									
		Wk 1		Wk 2		Wk 3		Wk 4		Wk 5		Wk 6		Wk 7		Wk 8		Wk 9		Wk 10		Wk 11		Wk 12			
		D 1	D 4	D 8	D 11	D 15	D 18	D 22	D 25	D 29	D 32	D 36	D 39	D 43	D 46	D 50	D 53	D 57	D 60	D 64	D 67	D 71	D 74	D 78	D 81		
PK/PD Sample (Escalation Only)		X[13]	X[13]	X[14]	X[14]	X[14]	X[14]	X[14]	X[14]							X[15]											
PK Sample (5 Expansion Pts)		X[13]	X[13]														X[16]										
PD Sample (All Expansion Pts)		X[16]	X[16]														X[16]										
Tumor Biopsy (Escalation Only)[11,]	X								X[11]																		
Tumor Biopsy (Expansion Only)[18]	X													X[18]													
CD36 testing (CD36-high cohort)[19]	X													X[18]													
Tumor Biomarkers[20]	X																X									X	
Reflex Testing[21]		To be performed for any patient who experiences a ≥Grade 2 IRR after administration of any study drug.																									

1. No study-directed procedures should be performed prior to obtaining written informed consent.
2. The medical history should be pertinent to the needs of the study and focused on the underlying malignancy and other ongoing medical conditions.
3. Vital signs will include pulse, blood pressure, respiratory rate, temperature and weight.
4. ECGs should be performed in triplicate at approximately 5-minute intervals (±5 minutes). ECGs to be obtained pre-infusion and at the conclusion of the VT1021 infusion.
5. Complete blood count to include total WBC, hemoglobin, hematocrit, platelet count, and WBC differential.
6. Serum chemistries to include sodium, potassium, chloride, bicarbonate, glucose, BUN, serum creatinine, AST, ALT, alkaline phosphatase, total bilirubin, calcium, phosphorous, magnesium, total protein, albumin, uric acid, lipase and amylase. After the completion of the 2nd cycle, and at the discretion of the Clinical Investigator, Day 8 and 15 serum chemistry testing is optional.
7. Coagulation parameters to include aPTT/PT/INR. A prothrombin time is NOT required if the institution's laboratory only reports out an INR. After baseline, coagulation studies are to be repeated as clinically indicated.
8. Urinalysis include dipstick determination of specific gravity, pH, protein, glucose, ketones, bilirubin, hemoglobin or occult blood; microscopic exam should be performed if the protein or hemoglobin/blood determinations are 2+ or higher.

9. Serum or urine pregnancy testing will be required monthly.
10. Radiographic imaging (CT or MRI) for RECIST/iRECIST (or RANO for GBM patients) assessment of disease. Tumors will be assessed by CT or MRI at Screening to establish extent of disease with imaging of sites to include chest, abdomen, and pelvis as well as other sites appropriate to the patient's disease status. GBM patients will require CT and / or MRI of the head and other sites as appropriate to the patient's disease status. The baseline imaging technique and sites imaged should be used for follow up scans while the patient is on study. Tumor response will be evaluated every 8 weeks ± 1 week.
11. If a Screening tumor biopsy cannot be obtained, submission of archival tissue is strongly recommended. On-study biopsy samples are strongly recommended during Cycle 1 Week 4 or any time during Cycle 2. However, biopsy may be obtained after Cycle 2 at the discretion of the Investigator. Biopsy will be taken on the day of infusion or within 24 hrs of infusion.
12. Brief targeted physical examinations focused on areas involved by adverse events or areas of tumor involvement.
13. Blood sample collection (for PK and PD analysis) will be obtained (± 10 minutes) pre-dose, 0, 2, 4, and 6 hours (and 24 hours OPTIONAL, ± 1 hour, Dose Escalation Phase only) after completion of the infusion after each dose in Cycle 1 Week 1 (C1D1 and C1D4).
14. Blood sample collection (for PK and PD analysis) will be obtained (± 10 minutes) pre-dose, 0, and 2 hours after completion of the infusion after each dose in Cycle 1 Week 2, 3 and 4 (C1D8, C1D11, C1D15, C1D18, C1D22, C1D25).
15. Blood sample collection (for PK analysis) will be obtained (± 10 minutes) pre-dose, 0, 2, 4, and 6 hours (and 24 hours OPTIONAL, ± 1 hour, Dose Escalation Phase only) after completion of the infusion after the first dose in Cycle 2 Week 8 (C2D50).
16. Blood sample collection (for PK and PD analysis) will be obtained (± 10 minutes) pre-dose, 0, 2, 4, and 6 hours after completion of the infusion
17. All procedures and evaluations required at the 30-day follow-up visit should also be completed as soon as possible after a patient discontinues study drug. Patients will then enter a follow-up period of 30 days after which patients be assessed again to assure patient safety.
18. If a Screening tumor biopsy cannot be obtained, submission of archival tissue collected within 6 months is required. For both Screening and archival tumor samples a formalin fixed paraffin embedded (FFPE) biopsy block is preferred. If a tissue block is not available for the pre-study tumor sample then slides should be submitted, see the laboratory manual for details. An on-study tumor biopsy sample, excluding Dose Expansion Cohort D (GBM), is required during Cycle 2 after C2D43. However, biopsy may be obtained after Cycle 2 at the discretion of the Investigator. Biopsy must be collected on the day of infusion after the infusion occurs or within 24 hrs of infusion the following day.
19. Screening tumor biopsy samples from patients under consideration for enrollment in Cohort E will be evaluated for expression of CD36 by immunohistochemistry (IHC), as determined by a scoring index. Patients with solid tumors with high expression of CD36 will be permitted to enroll in Cohort E. CD36 testing by IHC will also be performed on all pre- and on-study tumor biopsy samples from all other cohorts (excluding Dose Expansion Cohort D- on-study tumor biopsies for GBM are not required) as a pharmacodynamic parameter. For any tumor biopsies (archival or fresh), an FFPE biopsy block is preferred.
20. If applicable and when available, the results of previously obtained tumor biomarkers should be provided at Screening for Dose Expansion Cohorts. Biomarkers may include BRCA mutation status (positive or negative), MGMT methylation status (positive or negative), TMB score (number of mutations per megabase), MMR deficiency (positive or negative), microsatellite stability status (MSS or MSI; if MSI then low or high), and PD-L1 status (positive or negative). A blood sample for measurement of other tumor biomarkers will be evaluated during Screening, then every 8 weeks ± 1 week in association with tumor scans, and at the EOS visit (Dose Expansion Cohorts only). Samples

will be assessed locally. Tumor biomarkers to be measured for specific indications in Dose Expansion Cohorts include: Ovarian: CA125, Pancreatic: CA-19-9, TNBC: CA 15-3, Basket high-CD36: appropriate biomarker per indication.

21. Reflex testing includes blood sample collection for hematology, clinical chemistry, complement panel (CH50, C3a, C5a), tryptase, PK, and inflammatory cytokines.

Appendix 2: The ECOG and Karnofsky Performance Scores

Karnofsky Status Description	Karnofsky Grade	ECOG Grade	ECOG Status Description
Normal, no complaints.	100	0	Normal activity. Fully active, able to carry on all pre-disease performance without restriction.
Able to carry on normal activities. Minor signs of symptoms of disease.	90	1	Symptoms, but ambulatory. Restricted in physically strenuous activity, but ambulatory and able to carry out work of a light or sedentary nature (e.g., light housework, office work).
Normal activity with effort.	80	1	Symptoms, but ambulatory. Restricted in physically strenuous activity, but ambulatory and able to carry out work of a light or sedentary nature (e.g., light housework, office work).
Care for self. Unable to carry on normal activity or to do active work.	70	2	In bed < 50% of the time. Ambulatory and capable of all self-care, but unable to carry out any work activities. Up and about more than 50% of waking hours.
Requires occasional assistance, but able to care for most of his needs.	60	2	In bed < 50% of the time. Ambulatory and capable of all self-care, but unable to carry out any work activities. Up and about more than 50% of waking hours.
Requires considerable assistance and frequent medical care.	50	3	In bed > 50% of the time. Capable of only limited self-care, confined to bed or chair more than 50% of waking hours.
Disabled. Requires special care and assistance.	40	3	In bed > 50% of the time. Capable of only limited self-care, confined to bed or chair more than 50% of waking hours.
Severely disabled. Hospitalization indicated though death nonimminent.	30	4	100% bedridden. Completely disabled. Cannot carry on any self-care. Totally confined to bed or chair.
Very sick. Hospitalization necessary. Active supportive treatment necessary.	20	4	100% bedridden. Completely disabled. Cannot carry on any self-care. Totally confined to bed or chair.

Moribund	10	4	100% bedridden. Completely disabled. Cannot carry on any self-care. Totally confined to bed or chair.
Dead	0	5	Dead

Adapted from Oken MM, Creech RH, Tormey DC et al. Toxicity and response criteria of the Eastern Cooperative Oncology Group. Am J Clin Oncol 1982; 5:649-55.

Appendix 3: RECIST 1.1 Criteria

Adapted from E.A. Eisenhauer, et al: New response evaluation criteria in solid tumors: Revised RECIST guideline (version 1.1). European Journal of Cancer 45 (2009) 228–247

CATEGORIZING LESIONS AT BASELINE

Measurable Lesions

Lesions that can be accurately measured in at least one dimension.

- Lesions with longest diameter twice the slice thickness and at least 10 mm or greater when assessed by CT or MRI (slice thickness 5-8 mm)
- Lesions with longest diameter at least 20 mm when assessed by Chest X-ray
- Superficial lesions with longest diameter 10 mm or greater when assessed by caliper
- Malignant lymph nodes with the short axis 15 mm or greater when assessed by CT.

  NOTE: The shortest axis is used as the diameter for malignant lymph nodes, longest axis for all other measurable lesions.

Non-measurable disease

Non-measurable disease includes lesions too small to be considered measurable (including nodes with short axis between 10 and 14.9 mm) and truly non-measurable disease such as pleural or pericardial effusions, ascites, inflammatory breast disease, leptomeningeal disease, lymphangitic involvement of skin or lung, clinical lesions that cannot be accurately measured with calipers, abdominal masses identified by physical exam that are not measurable by reproducible imaging techniques.

- Bone disease**:** Bone disease is non-measurable with the exception of soft tissue components that can be evaluated by CT or MRI and meet the definition of measurability at baseline.
- Previous local treatment: A previously irradiated lesion (or lesion subjected to other local treatment) is non-measurable unless it has progressed since completion of treatment.

Normal sites

- Cystic lesions**:** Simple cysts should not be considered as malignant lesions and should not be recorded either as target or non-target disease. Cystic lesions thought to represent cystic metastases can be measurable lesions, if they meet the specific definition above. If non-cystic lesions are also present, these are preferred as target lesions.
- Normal nodes**:** Nodes with short axis <10 mm are considered normal and should not be recorded or followed either as measurable or non-measurable disease.

RECORDING TUMOR ASSESSMENTS

All sites of disease must be assessed at baseline. Baseline assessments should be done as close as possible prior to study start. For an adequate baseline assessment, all required scans must be done within 28 days prior to study drug and all disease must be documented appropriately. If baseline assessment is inadequate, subsequent statuses generally should be indeterminate.

<u>Target lesions</u>

All measurable lesions up to a maximum of 2 lesions per organ, 5 lesions in total, representative of all involved organs, should be identified as target lesions at baseline. Target lesions should be selected on the basis of size (longest lesions) and suitability for accurate repeated measurements. Record the longest diameter for each lesion, except in the case of pathological lymph nodes for which the short axis should be recorded. The sum of the diameters (longest for non-nodal lesions, short axis for nodal lesions) for all target lesions at baseline will be the basis for comparison to assessments performed on study.

- If two target lesions coalesce the measurement of the coalesced mass is used. If a large target lesion splits, the sum of the parts is used.
- Measurements for target lesions that become small should continue to be recorded. If a target lesion becomes too small to measure, 0 mm should be recorded if the lesion is considered to have disappeared; otherwise a default value of 5 mm should be recorded.

  NOTE: When nodal lesions decrease to <10 mm (normal), the actual measurement should still be recorded.

<u>Non-target disease</u>

All non-measurable disease is non-target. All measurable lesions not identified as target lesions are also included as non-target disease. Measurements are not required but rather assessments will be expressed as ABSENT, INDETERMINATE, PRESENT/NOT INCREASED, INCREASED. Multiple non-target lesions in one organ may be recorded as a single item on the case report form (e.g., 'multiple enlarged pelvic lymph nodes' or 'multiple liver metastases').

OBJECTIVE RESPONSE STATUS AT EACH EVALUATION.

Disease sites must be assessed using the same technique as baseline, including consistent administration of contrast and timing of scanning. If a change needs to be made the case must be discussed with the radiologist to determine if substitution is possible. If not, subsequent objective statuses are indeterminate.

<u>Target disease</u>

- Complete Response (CR): Complete disappearance of all target lesions with the exception of nodal disease. All target nodes must decrease to normal size (short axis < 10 mm). All target lesions must be assessed.
- Partial Response (PR): Greater than or equal to 30% decrease under baseline of the sum of diameters of all target measurable lesions. The short diameter is used in the sum for target nodes, while the longest diameter is used in the sum for all other target lesions. All target lesions must be assessed.
- Stable: Does not qualify for CR, PR or Progression. All target lesions must be assessed. table can follow PR only in the rare case that the sum increases by less than 20% from the nadir, but enough that a previously documented 30% decrease no longer holds.
- Objective Progression (PD): 20% increase in the sum of diameters of target measurable lesions above the smallest sum observed (over baseline if no decrease in the sum is observed during therapy), with a minimum absolute increase of 5 mm.
- Indeterminate. Progression has not been documented, and
  - one or more target measurable lesions have not been assessed
  - or assessment methods used were inconsistent with those used at baseline

- or one or more target lesions cannot be measured accurately (e.g., poorly visible unless due to being too small to measure)
- or one or more target lesions were excised or irradiated and have not reappeared or increased.

Non-target disease

- CR: Disappearance of all non-target lesions and normalization of tumor marker levels. All lymph nodes must be 'normal' in size (<10 mm short axis).
- Non-CR/Non-PD: Persistence of any non-target lesions and/or tumor marker level above the normal limits.
- PD: Unequivocal progression of pre-existing lesions. Generally, the overall tumor burden must increase sufficiently to merit discontinuation of therapy. In the presence of SD or PR in target disease, progression due to unequivocal increase in non-target disease should be rare.
- Indeterminate: Progression has not been determined and one or more non-target sites were not assessed or assessment methods were inconsistent with those used at baseline.

New Lesions

The appearance of any new unequivocal malignant lesion indicates PD. If a new lesion is equivocal, for example due to its small size, continued assessment will clarify the etiology. If repeat assessments confirm the lesion, then progression should be recorded on the date of the initial assessment. A lesion identified in an area not previously scanned will be considered a new lesion.

Supplemental Investigations

- If CR determination depends on a residual lesion that decreased in size but did not disappear completely, it is recommended the residual lesion be investigated with biopsy or fine needle aspirate. If no disease is identified, objective status is CR.
- If progression determination depends on a lesion with an increase possibly due to necrosis, the lesion may be investigated with biopsy or fine needle aspirate to clarify status.

Subjective progression

Patients requiring discontinuation of study drug without objective evidence of disease progression should not be reported as PD on tumor assessment CRFs. This should be indicated on the end of study CRF as off study drug due to Global Deterioration of Health Status. Every effort should be made to document objective progression even after discontinuation of study drug.

Objective Response Status at each Evaluation

**Target Lesions**	**Non-target Disease**	**New Lesions**	**Objective status**
CR	CR	No	CR
CR	Non-CR/Non-PD	No	PR
CR	Indeterminate or Missing	No	PR
PR	Non-CR/Non-PD, Indeterminate, or Missing	No	PR
SD	Non-CR/Non-PD, Indeterminate, or Missing	No	Stable
Indeterminate or Missing	Non-PD	No	Indeterminate
PD	Any	Yes or No	PD
Any	PD	Yes or No	PD
Any	Any	Yes	PD

If the protocol allows enrollment of patients with only non-target disease, the following table will be used:

Objective Response Status at each Evaluation for Patients with Non-Target Disease Only

**Non-target Disease**	**New Lesions**	**Objective status**
CR	No	CR
Non-CR/Non-PD	No	Non-CR/Non-PD
Indeterminate	No	Indeterminate
Unequivocal progression	Yes or No	PD
Any	Yes	PD

Appendix 4: Description of the iRECIST Process for Assessment of Disease Progression

Assessment at Screening and Prior to RECIST 1.1 Progression

Until radiographic progression based on RECIST 1.1, there is no distinct iRECIST assessment.

Assessment and Decision at RECIST 1.1 Progression

In patients who show evidence of radiological PD by RECIST 1.1, the Investigator will decide whether to continue a patient on study drug until repeat imaging is obtained. This decision by the Investigator should be based on the patient's overall clinical condition.

Clinical stability is defined as the following:

- Absence of symptoms and signs indicating clinically significant progression of disease
- No decline in ECOG performance status
- No requirements for intensified management, including increased analgesia, radiation, or other palliative care

Any patient deemed clinically unstable should be discontinued from study drug at site-assessed first radiologic evidence of PD, and is not required to have repeat tumor imaging for confirmation of PD by iRECIST.

If the Investigator decides to continue study drug, the patient may continue to receive study drug and the tumor assessment should be repeated 4 to 8 weeks later to confirm PD by iRECIST, per Investigator assessment.

Tumor flare may manifest as any factor causing radiographic progression per RECIST 1.1, including:

- Increase in the sum of diameters of target lesion(s) identified at baseline to ≥ 20% and ≥ 5 mm from nadir
    - Please note: the iRECIST publication uses the terminology "sum of measurements", but "sum of diameters" will be used in this protocol, consistent with the original RECIST 1.1 terminology.
- Unequivocal progression of non-target lesion(s) identified at baseline
- Development of new lesion(s)

iRECIST defines new response categories, including iUPD (unconfirmed progressive disease) and iCPD (confirmed progressive disease). For purposes of iRECIST assessment, the first visit showing progression according to RECIST 1.1 will be assigned a visit (overall) response of iUPD, regardless of which factors caused the progression.

At this visit, target and non-target lesions identified at baseline by RECIST 1.1 will be assessed as usual.

New lesions will be classified as measurable or non-measurable, using the same size thresholds and rules as for baseline lesion assessment in RECIST 1.1. From measurable new lesions, up to 5 lesions total (up to 2 per organ), may be selected as New Lesions – Target. The sum of diameters

of these lesions will be calculated, and kept distinct from the sum of diameters for target lesions at baseline. All other new lesions will be followed qualitatively as New Lesions – Non-target.

Assessment at the Confirmatory Imaging

On the confirmatory imaging, the patient will be classified as progression confirmed (with an overall response of iCPD), or as showing persistent unconfirmed progression (with an overall response of iUPD), or as showing disease stability or response (iSD/iPR/iCR).

Confirmation of Progression

Progression is considered confirmed, and the overall response will be iCPD, if ANY of the following occurs:

- Any of the factors that were the basis for the initial iUPD show worsening
    - For target lesions, worsening is a further increase in the sum of diameters of ≥ 5 mm, compared to any prior iUPD time point
    - For non-target lesions, worsening is any significant growth in lesions overall, compared to a prior iUPD time point; this does not have to meet the "unequivocal" standard of RECIST 1.1
    - For new lesions, worsening is any of these:
        - An increase in the new lesion sum of diameters by ≥ 5 mm from a prior iUPD time point
        - Visible growth of new non-target lesions
        - The appearance of additional new lesions
- Any new factor appears that would have triggered PD by RECIST 1.1

Persistent iUPD

Progression is considered not confirmed, and the overall response remains iUPD, if:

- None of the progression-confirming factors identified above occurs AND
- The target lesion sum of diameters (initial target lesions) remains above the initial PD threshold (by RECIST 1.1)

Additional imaging for confirmation should be scheduled 4 to 8 weeks from the scan on which iUPD is seen. This may correspond to the next visit in the original visit schedule. The assessment of the subsequent confirmation scan proceeds in an identical manner, with possible outcomes of iCPD, iUPD, and iSD/iPR/iCR.

Resolution of iUPD

Progression is considered not confirmed, and the overall response becomes iSD/iPR/iCR, if:

- None of the progression-confirming factors identified above occurs, AND
- The target lesion sum of diameters (initial target lesions) is not above the initial PD threshold.

The response is classified as iSD or iPR (depending on the sum of diameters of the target lesions), or iCR if all lesions resolve.

In this case, the initial iUPD is considered to be pseudo-progression, and the level of suspicion for progression is “reset”. This means that the next visit that shows radiographic progression, whenever it occurs, is again classified as iUPD by iRECIST, and the confirmation process is repeated before a response of iCPD can be assigned.

Management Following the Confirmatory Imaging

If repeat imaging does not confirm PD per iRECIST, as assessed by the Investigator, and the patient continues to be clinically stable, study drug may continue and follow the regular imaging schedule. If PD is confirmed, patients will be discontinued from study drug.

NOTE: If a patient has confirmed radiographic progression (iCPD) as defined above, but the patient is achieving a clinically meaningful benefit, an exception to continue study drug may be considered. In this case, if study drug is continued, tumor imaging should continue to be performed.

Detection of Progression at Visits After Pseudo-progression Resolves

After resolution of pseudo-progression (i.e., achievement of iSD/iPR/iCR), iUPD is indicated by any of the following events:

- Target lesions
    - Sum of diameters reaches the PD threshold (≥ 20% and ≥ 5 mm increase from nadir) either for the first time, or after resolution of previous pseudo-progression. The nadir is always the smallest sum of diameters seen during the entire study, either before or after an instance of pseudo-progression.
- Non-target lesions
    - If non-target lesions have never shown unequivocal progression, their doing so for the first time results in iUPD.
    - If non-target lesions had shown previous unequivocal progression, and this progression has not resolved, iUPD results from any significant further growth of non-target lesions, taken as a whole.
- New lesions
    - New lesions appear for the first time
    - Additional new lesions appear
    - Previously identified new target lesions show an increase of ≥ 5 mm in the new lesion sum of diameters, from the nadir value of that sum
    - Previously identified non-target lesions show any significant growth

If any of the events above occur, the overall response for that visit is iUPD, and the iUPD evaluation process (see Assessment at the Confirmatory Imaging above) is repeated. Progression must be confirmed before iCPD can occur.

The decision process is identical to the iUPD confirmation process for the initial PD, except in one respect. If new lesions occurred at a prior instance of iUPD, and at the confirmatory scan the burden of new lesions has increased from its smallest value (for new target lesions, their sum of diameters is ≥5 mm increased from its nadir), then iUPD cannot resolve to iSD or iPR. It will remain iUPD until either a decrease in the new lesion burden allows resolution to iSD or iPR, or until a confirmatory factor causes iCPD.

Additional details about iRECIST are provided in the iRECIST publication (Seymour et al, 2017).

Appendix 5: CTCAE v5.0 Criteria

(https://ctep.cancer.gov/protocoldevelopment/electronic_applications/docs/CTCAE_v5_Quick_Reference_5x7.pdf)

Appendix 6: The Declaration of Helsinki

https://www.wma.net/policies-post/wma-declaration-of-helsinki-ethical-principles-for-medical-research-involving-human-subjects/

Appendix 7: The RANO Criteria for Assessment of Glioblastoma Patients

1. MEASUREMENT OF EFFECT

This study will utilize the criteria proposed by the Response Assessment in Neuro-Oncology (RANO) working group (Wen et al., 2010).

Radiologic assessment will be determined by the Response Assessment in Neuro-Oncology Working Group (RANO) Criteria using bidirectional tumor measurements with some modifications. In addition to imaging characteristics, these criteria include consideration of neurological function and corticosteroid use (Wen et al., 2010). The RANO Criteria is outlined in detail in this section.

Magnetic resonance imaging (MRI) is the most readily available and reproducible method of disease assessment and is required for this study. The largest and most representative lesions should be measured either on axial, coronal or sagittal slices, and chosen to be followed for response evaluation.

The recommended sequences are outlined in detail below and should conform as closely as possible to the consensus recommendations for a standardized Brain Tumor Imaging Protocol in clinical studies.

1.1 Antitumor Effect - Definitions

Evaluable for toxicity. All participants who receive at least one dose of study drug will be evaluable for toxicity from the time of their first drug.

Evaluable for objective response. Only those participants who have measurable disease present at baseline (Cycle 1, Day 1 scan) and have received at least one dose of therapy will be considered evaluable for response. These participants will have their response classified according to the definitions stated below. (Note: Participants who exhibit objective disease progression or die prior to the end of cycle 1 will also be considered evaluable.)

Measurable disease. Bi-dimensionally, contrast-enhancing, measurable lesions with clearly defined margins by CT or MRI scan, with a minimal diameter of 1 cm, and visible on 2 axial slices which are at least 5 mm apart with 0 mm skip. Measurement of tumor around a cyst or surgical cavity, if necessary, requires a minimum thickness of 3 mm. If there are too many measurable lesions to measure at each evaluation, the Investigator must choose the largest two to be followed before a participant is entered on study. The remaining lesions will be considered non-measurable for the purpose of objective response determination. Unless progression is observed, objective response can only be determined when all measurable and non-measurable lesions are assessed.

Non-measurable evaluable disease. Uni-dimensionally measurable lesions, masses with margins not clearly defined, lesions with maximal diameter < 1cm.

1.2 Response/Progression Categories. This will eliminate the use of FLAIR in determining progression per section 1.5

Complete response (CR). All of the following criteria must be met:

a) Complete disappearance of all enhancing measurable and non-measurable disease sustained for at least 4 weeks. In the absence of a confirming scan 4 weeks later, this scan will be considered only stable disease.
b) No new lesions.
c) All measurable and non-measurable lesions must be assessed using the same techniques as baseline.
d) Participants must be on no steroids or on physiologic replacement doses only.
e) Stable or improved clinically, for clinical signs and symptoms present at baseline and recorded to be disease related.

Participants with non-measurable disease cannot have a complete response. The best response possible is stable disease.

Partial response (PR). All of the following criteria must be met:

a) Greater than or equal to 50% decrease compared to baseline in the sum of products of perpendicular diameters of all measurable enhancing lesions sustained for at least 4 weeks. In the absence of a confirming scan 4 weeks later, this scan will be considered only stable disease.
b) No progression of non-measurable disease.
c) No new lesions.
d) All measurable and non-measurable lesions must be assessed using the same techniques as baseline.
e) The steroid dose at the time of the scan evaluation should be no greater than the dose at time of baseline scan.
f) Stable or improved, for clinical signs and symptoms present at baseline and recorded to be disease related clinically.

Participants with non-measurable disease cannot have a partial response. The best response possible is stable disease.

Progressive disease (PD). The following criterion must be met:

a) > 25% increase in sum of the products of perpendicular diameters of enhancing lesions (over best response or baseline if no decrease) on stable or increasing doses of corticosteroids

and/or one or more of the of the following:

b) Any new lesion.
c) Clear clinical deterioration not attributable to other causes apart from the tumor (e.g., seizures, medication side effects, complications of therapy, cerebrovascular events, infection, etc.). The definition of clinical deterioration is left to the discretion of the Investigator but it is recommended that a decline in the Karnofsky Performance Score (KPS) from 100 or 90 to 70 or less, a decline in KPS of at least 20 from 80 or less, or a decline in KPS from any baseline to 50 or less, for at least 7 days, be considered neurologic deterioration, unless attributable to co-morbid

events or changes in corticosteroid dose.

d) Failure to return for evaluation due to death or deteriorating condition.

Stable disease (SD). All of the following criteria must be met:

a) Does not qualify for CR, PR, or progression.
b) All measurable and non-measurable sites must be assessed using the same techniques as baseline.
c) Stable clinically.

Unknown response status. Progressive disease has not been documented and one or more measurable or non-measurable lesions have not been assessed.

These RANO Response Criteria are also summarized in Table AA:

Table AA: Summary of the RANO Response Criteria

	CR	PR	SD	PD#
T1-Gd +	None	≥50% decrease	<50% decrease-<25% increase	≥25% increase*
New Lesion	None	None	None	Present*
Corticosteroids	None	Stable or decrease	Stable or decrease	NA
Clinical Status	Stable or increase	Stable or increase	Stable or increase	Decrease*
Requirement for Response	All	All	All	Any*

CR=complete response; PR=partial response; SD=stable disease; PD=progressive disease
#: Progression occurs when any of the criteria with * is present
NA: Increase in corticosteroids alone will not be taken into account in determining progression in the absence of persistent clinical deterioration

1.3 Methods for Evaluation of Measurable Disease

All measurements should be taken and recorded in metric notation, using a ruler, calipers, or digital measurement tool. All baseline evaluations should be performed as closely as possible to the beginning of study drug and never more than 14 days from the date of registration.

The same method of assessment and the same technique should be used to characterize each identified and reported lesion at baseline and during follow up.

1.4 Evaluation of Best Response

The best overall response is the best response recorded from the start of study drug until disease progression (taking as reference for progressive disease the smallest measurements recorded since the study drug started). If a response recorded at one scheduled MRI does not persist at the next regular scheduled MRI, the response will still be recorded based on the prior scan but will

be designated as a non-sustained response. If the response is sustained, i.e., still present on the subsequent MRI, it will be recorded as a sustained response, lasting until the time of tumor progression. Participants without measurable disease may only achieve SD or PD as their best "response."

1.5 Modified RANO (iRANO): Study Continuation Beyond Initial Progressive Disease (Okada et al. 2015)

Immunotherapeutic agents such as pembrolizumab may produce antitumor effects by potentiating endogenous cancer-specific immune responses which may manifest as initial worsening of enhancement and edema on MRI or CT scans (i.e., pseudoprogression). In addition, the response patterns seen with immunotherapeutics may extend beyond the typical time course of responses seen with cytotoxic agents and can manifest a clinical response after an initial increase in tumor burden or even the appearance of new lesions. For these reasons, the immune-related response criteria (irRC) have endorsed continuation of study therapy beyond initial radiographic evidence of progression for clinically stable patients undergoing immune based therapies (Wolchok et al. 2009).

A major advance of the RANO criteria (Wen et al. 2010) to assess response in neuro-oncology over the previously used Macdonald criteria (Macdonald et al. 1990) includes recognition of the prevalence of pseudoprogression during the first three months following completion of radiation and daily temozolomide (Brandes et al. 2008, Brandsma et al. 2008). Specifically, RANO permits patients with such progressive MRI findings to continue temozolomide therapy for up to three months in order to avoid inaccurately classifying such patients as progressive. Furthermore, RANO permits patients with progressive radiographic findings at any time to continue current therapy pending follow-up imaging if the etiology of progressive imaging findings is unclear. Standard RANO may not provide an accurate response assessment of immunotherapeutic agents such as pembrolizumab.

Therefore, the following adaptations of the RANO criteria will be used to assess response for patients treated on this study in an exploratory fashion (Table BB):

- Potential Pseudoprogression: If radiologic imaging shows initial PD, patients who are not experiencing significant clinical decline (e.g., significant decrease in KPS, may be allowed to continue study drug for up to three months. Patients should be closely monitored with MRIs every cycle (approximately every 4 weeks) during this period. Patients who have radiographic evidence of further progression after up to three months, or who decline significantly at any time, will be classified as progressive with the date of disease progression back-dated to the first date that the patient met criteria for progression and such patients will be discontinued from study therapy. Although the kinetics of pseudoprogression due to immune checkpoint blockade among glioblastoma patients is currently unknown, three months is a reasonable estimate based on: 1) the peak time for XRT/daily temozolomide-related pseudoprogression is usually within three months of completion for glioblastoma patients (Brandes et al. 2008, Brandsma et al. 2008) and; 2) three months is also the most common timeframe for pseudoprogression observed among patients with advanced melanoma or other solid tumors treated with PD-1/PD-L1 immune checkpoint blockade to date (Brahmer et al. 2012, Hamid et al. 2013, Topalian et al. 2012).

Among patients on this study with initial radiographic PD, tumor assessment should be repeated regularly (every cycle, approximately every 4 weeks) in order to confirm PD with the option of continuing study drug as described below while awaiting radiologic confirmation of progression. If repeat imaging shows a stabilization or reduction in the tumor burden compared to the initial scan demonstrating PD, study drug may be continued / resumed. If repeat imaging after up to three months confirms progressive disease, then the date of disease progression will be the first date the patient met criteria for progression and patients will be discontinued from study therapy. Patients who have confirmed disease progression will discontinue study medication and enter the follow-up/survival phase of the study. In determining whether or not the tumor burden has increased or decreased, Investigators should consider all target lesions as well as non-target lesions.

- Tumor Enhancement to Define Progression: RANO expanded the previously utilized Macdonald criteria (Macdonald et al. 1990) to include the development of "significantly" increased T2 or FLAIR abnormality in the definition of progressive disease because such changes can be a major component defining radiographic progression following therapeutic use of VEGF/VEGFR-targeting therapeutics which are known to elicit potent anti-permeability changes that limit contrast uptake. However, immune based therapies are expected to be associated with inflammatory changes that may include edema. Therefore, radiographic progressive disease will be defined by assessment of enhancing tumor and will not declare tumor progression based on the presence of T2 or FLAIR changes alone as outlined in RANO because:
    - There is no expectation that immunotherapy agents including PD-1 inhibitors will falsely diminish enhancing tumor burden as has been noted with anti-angiogenic therapies; and
    - Immune based therapies are expected to induce inflammatory responses which may be associated with increased edema and T2/FLAIR changes. Such radiographic finding may inaccurately be interpreted to represent tumor progression (i.e., pseudoprogression).

In patients who have initial evidence of radiographic PD, it is at the discretion of the treating physician whether to continue a patient on study drug for up to three months pending confirmation of PD on follow-up imaging. This clinical judgment decision should be based on the patient's overall clinical condition, including performance status, clinical symptoms, and laboratory data. Patients may receive study drug while waiting for confirmation of PD if they are not experiencing significant clinical decline and the patient is adequately tolerating study therapy (if a patient is required to discontinue study drug for toxicity as defined per Section 7.7, then they must be taken off study drug).

When feasible, study therapy should not be discontinued until radiographic progression is confirmed. This allowance to continue study drug despite initial radiologic progression takes into account the observation that some patients can have a transient tumor flare in the first few months after the start of immunotherapy, but with subsequent disease response (Wolchok et al. 2009). Patients that are exhibiting significant neurologic decline

are not required to have repeat imaging for confirmation of progressive disease.

Table BB: Imaging and Treatment After 1st Radiologic Evidence of PD

	No Significant Neurologic Decline		Significant Neurologic Decline	
	**Imaging**	**Treatment**	**Imaging**	**Treatment**
1st radiologic evidence of PD	Repeat imaging (every cycle, approximately every 6 weeks) for up to 3 months to confirm PD	May continue study drug at the Investigator’s discretion for up to 3 months while awaiting confirmatory scans	Repeat imaging > 6 weeks later to confirm PD if possible	Discontinue study drug
Repeat scan up to 3 months after 1st radiologic evidence confirms PD	No additional imaging required; date of tumor progression back-dated to date of initial radiographic PD	Discontinue study drug	No additional imaging required	Not applicable
Repeat scan shows SD, PR or CR	Continue regularly scheduled imaging assessments every 6 weeks	Continue study drug at the Investigator’s discretion	Continue regularly scheduled imaging assessments every 6 weeks	May restart study drug if condition has improved and/or clinically stable per Investigator’s discretion

Participants with progressive radiographic findings are encouraged to undergo surgical intervention in order to delineate pseudoprogression due to inflammation associated with study drug from true tumor progression. Participants with histopathologic findings of significant immune infiltrate and evolving gliosis will be allowed to continue study therapy. In contrast, those with clear evidence of progressive tumor by histopathologic evaluation will be defined as progressive and discontinued from study therapy. For such patients, the date of tumor progression will be the first date the participant met radiographic criteria for PD.

Appendix 7 References

Brahmer JR, Tykodi SS, Chow LQ, et al. Safety and activity of anti-PD-L1 antibody in patients with advanced cancer. N Engl J Med. 2012; 366(26):2455-2465.

Brandes AA, Tosoni A, Spagnolli F, et al. Disease progression or pseudoprogression after concomitant radiochemotherapy treatment: pitfalls in neurooncology. Neuro Oncol. 2008; 10(3):361-367.

Brandsma D, Stalpers L, Taal W, Sminia P, van den Bent MJ. Clinical features, mechanisms, and management of pseudoprogression in malignant gliomas. Lancet Oncol. 2008; 9(5):453-461.

Hamid O, Robert C, Daud A, et al. Safety and tumor responses with lambrolizumab (anti-PD-1) in melanoma. N Engl J Med. 2013; 369(2):134-144.

Macdonald DR, Cascino TL, Schold SC, Jr., Cairncross JG. Response criteria for phase II studies of supratentorial malignant glioma. J Clin Oncol. 1990; 8(7):1277-1280.

Okada H, Weller M, Huang R, et al. Immunotherapy Response Assessment in Neuro-Oncology (iRANO): A Report of the RANO Working Group. Lancet Oncol. 2015; 16(15):e534-e542.

Topalian SL, Hodi FS, Brahmer JR, et al. Safety, activity, and immune correlates of anti-PD-1 antibody in cancer. N Engl J Med. 2012; 366(26):2443-2454.

Wen PY, Macdonald DR, Reardon DA, et al. Updated response assessment criteria for high-grade gliomas: response assessment in neuro-oncology working group. J Clin Oncol. 2010; 28(11):1963-1972.

Wolchok JD, Hoos A, O'Day S, et al. Guidelines for the evaluation of immune therapy activity in solid tumors: immune-related response criteria. Am J Clin Cancer Res. 2009; 15(23):7412-7420.
